# N-terminal acetylation modulates peptide stability, fibrillogenesis and MMP-7 processing

**DOI:** 10.1007/s00253-026-14007-5

**Published:** 2026-08-15

**Authors:** Agnieszka Kubiś, Justyna Sawicka, Marcin Włodarczyk, Aleksandra Szwed-Georgiou, Mirosława Panasiuk, Natalia Karska, Beata Gromadzka, Przemysław Płociński, Sylwia Rodziewicz-Motowidło

**Affiliations:** 1 Department of Biomedical Chemistry, Faculty of Chemistry, University of Gdańsk, Wita Stwosza 63, Gdańsk, 80-308 Poland; 2 Department of In Vitro Studies, Institute of Biotechnology and Molecular Medicine, Kampinoska 25, Gdańsk, 80-180 Poland; 3 Department of Immunology and Infectious Biology, Faculty of Biology and Environmental Protection, University of Łódź, Banacha 12/16, Łódź, 90-237 Poland; 4 NanoExpo LTD, Kładki 24, Gdańsk, 80-822 Poland

**Keywords:** Self-assembling peptides, Peptide nanofibers, *N*-terminal acetylation, MMP-7-mediated processing, Osteoblast cytocompatibility, Fibrillar peptide assemblies

## Abstract

**Abstract:**

Short self-assembling peptides are versatile building blocks for enzyme-responsive nanostructured biomaterials. Here, we investigate how *N*-terminal acetylation affects the stability, fibrillogenesis, and protease-mediated fragment formation of two modular peptides, ug51 and ug52, composed of a fibrillogenic QAGIVV segment, an MMP-7-cleavable PLGL linker, and a *C*-terminal domain derived from motifs related to osteogenic growth peptides (OGPs). The stability of the peptides was assessed in water and cell culture medium, while secondary structure and nanoassembly were analyzed by circular dichroism, thioflavin T fluorescence, and transmission electron microscopy (TEM). Biological effects were evaluated in hFOB 1.19 osteoblasts. The non-acetylated peptide ug51 underwent spontaneous cleavage in the Val-Pro region, generating defined fragments, including PLGLYGFGG and, after prolonged incubation, the OGP-related LYGFGG sequence. In contrast, the *N*-terminally acetylated analog ug52 displayed markedly higher stability and formed ThT-positive, TEM-visible fibrillar assemblies. These assemblies remained susceptible to MMP-7-mediated processing, as shown by MALDI-TOF MS detection of the LYGFGG fragment after enzymatic incubation. Biological assays in hFOB 1.19 osteoblasts indicated overall cytocompatibility within the tested concentration range and peptide- and fragment-dependent effects on metabolic/proliferation-associated activity and migration-related responses. These findings suggest that *N*-terminal acetylation can shift the behavior of this modular peptide system from spontaneous degradation toward a more stable, fibril-forming, protease-processable state. Thus, this study provides proof-of-concept evidence that *N*-terminal acetylation can modulate the balance between peptide stability, supramolecular assembly, and enzymatic processability in a short modular peptide system.

**Key points:**

• *N-terminal acetylation increases the apparent stability of the modular peptide ug52.*

• *Non-acetylated ug51 undergoes spontaneous Val-Pro cleavage.*

• *Acetylated ug52 forms ThT/TEM-positive fibrils that remain MMP-7-processable.*

**Graphical abstract:**

**Figure:**
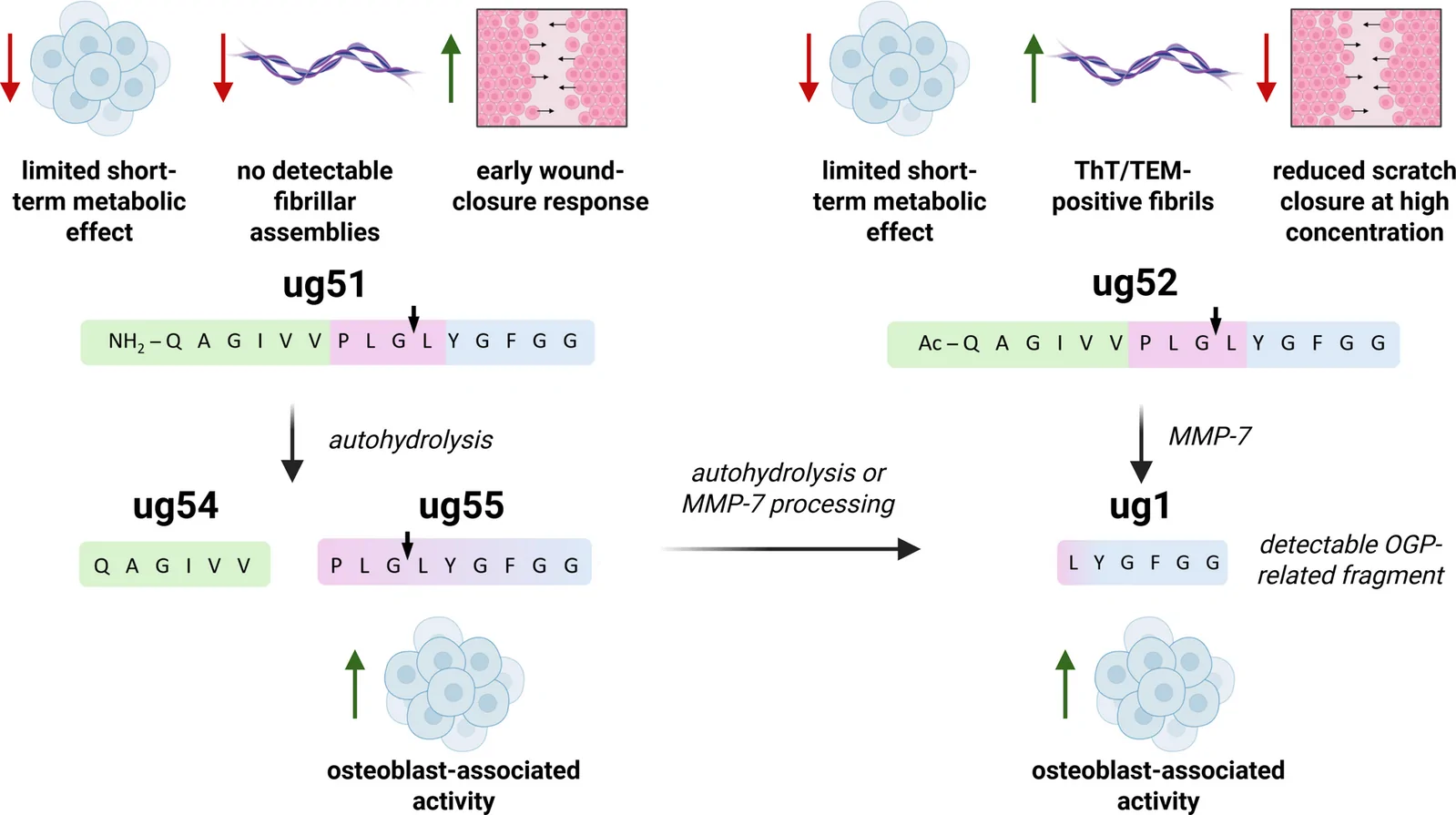

**Supplementary information:**

The online version contains supplementary material available at https://doi.org/10.1007/s00253-026-14007-5.

## Introduction

Short self-assembling peptides are emerging building blocks for peptide-based bionanoparticles and nanostructured biomaterials. Their ability to organize into β-sheet-rich fibrils can be used to create extracellular-matrix-like architectures and to present bioactive signals at high density. These fibrillogenic systems, spanning peptide amphiphiles and ionic-complementary sequences, have been tailored to support cell adhesion, migration, and tissue-specific signaling, making them attractive platforms in regenerative medicine (Hartgerink et al. 2001, 2002; Matson and Stupp 2011). Due to their nanoscale dimensions and ability to form an ordered supramolecular network, self-assembling peptide fibrils can also be considered a class of functional bionanoparticles, in which structural organization at the nanoscale enables both mechanical support and the presentation of bioactive motifs (Webber et al. 2016). Peptide fibrils can retain and release proteins and small molecules in a sustained manner, allowing the fibrillar network to function as a structural scaffold or as a reservoir for therapeutics and endogenous molecules (Schneider et al. 2008; Dzierżyńska et al. 2023; Sedighi et al. 2023). Classic examples of self-assembling peptide systems include peptide amphiphiles developed by Stupp and coworkers, ionic-complementary peptides such as RADA16, and designed β-sheet-forming sequences such as MAX peptides, all of which assemble into nanofibrillar scaffolds suitable for biomedical applications (Hartgerink et al. 2001; Deptuła et al. 2023). Proof-of-concept studies have shown controlled release from peptide hydrogels and accelerated wound re-epithelialization when growth factors are incorporated into self-assembling peptide matrices (Sedighi et al. 2023; Ligorio et al. 2024). A key factor influencing the behavior of therapeutic peptides and peptide-based materials is terminal modification, particularly *N*-terminal acetylation. In biological systems, *N*-terminal acetylation is among the most prevalent protein *N*-terminal modifications and can influence protein stability, molecular interactions, and subcellular localization (Wang et al. 2022; Weeks et al. 2022; Miłogrodzka et al. 2025). In synthetic peptides, capping the *N*-terminus alters the overall charge, hydrogen bonding, and steric environment at the peptide end; these are factors that can shift secondary-structure propensity and supramolecular assembly pathways. For example, spectroscopy on the fibrillizing peptide model KFE8 revealed that *N*-terminal acetylation measurably perturbs its hydrogen-bonding network and aggregation thermodynamics (Weeks et al. 2022). Similarly, systematic work on short glutamate oligomers showed that *N*-terminal acetylation and/or *C*-terminal amidation modulate amyloidogenicity and change fibril morphology, underscoring the sensitivity of assembly to end-group chemistry (Hernik-Magoń et al. 2017). Furthermore, terminal modifications can dictate the functional role of a peptide in aggregation pathways. For example, while *N*-formylation at the *N*-terminus of the bacterial peptide PSMα3 promotes its assembly in toxic cross-α fibrils, its synthetic deformylated analog (dfPSMα3) remains monomeric and acts as a selective inhibitor of human islet amyloid polypeptide (hIAPP) aggregation. The efficacy of this inhibition is notably dependent on the *C*-terminal state of the target protein, as dfPSMα3 fails to attenuate the fibrillation of deamidated hIAPP (Kalitnik et al. 2025). Peptide nanofibers and peptide hydrogels have also been explored as delivery systems for bioactive molecules, including growth factors, antimicrobial peptides, and small-molecule drugs (Huang et al. 2019; Gallo et al. 2021; Copling et al. 2023). In such systems, supramolecular fibrillar networks can immobilize cargo through covalent or non-covalent interactions and release it in response to enzymatic degradation or environmental stimuli. In this context, peptide nanofibers can serve as supramolecular reservoirs in which molecular organization, enzymatic susceptibility, and matrix interactions collectively influence the availability of embedded or covalently linked bioactive motifs (Oliveira et al. 2022; Yaylaci et al. 2023). Enzyme-responsive peptide assemblies represent a particularly attractive strategy in regenerative medicine, as protease-mediated cleavage can contribute to localized processing and potential generation of bioactive fragments within remodeling tissues (Ulijn and Smith 2008; Giano et al. 2011). In particular, MMP-cleavable peptide motifs have been incorporated into hydrogel networks to provide cell- or tissue-remodeling-responsive degradation and to support applications in bone regeneration and wound repair (Chen et al. 2021; Oliver-Cervelló et al. 2023; Sun et al. 2024). From a pharmacological perspective, *N*-terminal acetylation can also improve serum stability by reducing susceptibility to aminopeptidases, as demonstrated for M2-macrophage-targeting peptides where capped analogs resisted *N*-terminal trimming and performed better in vivo (Ngambenjawong et al. 2016). OGP and its C-terminal active fragment OGP(10–14) have been investigated as osteoblast-related peptide signals involved in bone formation and regeneration. OGP-derived motifs have also been incorporated into biomaterial systems to enhance osteoblast-associated responses, including cell proliferation, matrix formation, and osteogenic differentiation (Pigossi et al. 2016). Therefore, short OGP-related sequences provide a useful bioactive module for designing osteoblast-oriented peptide materials. Although assembly and bioactivity are central to function, chemical stability in aqueous media is an equally critical attribute for deployable peptide scaffolds and therapeutics.

Despite growing interest in self-assembling peptide nanomaterials, it remains insufficiently understood how terminal modification affects the interplay between chemical stability, supramolecular assembly, protease susceptibility, and biological response in short modular peptides. Importantly, N-terminal acetylation, self-assembling peptide fibrils, MMP-sensitive linkers, and OGP-related motifs have each been investigated previously; therefore, the novelty of the present study does not lie in any single component alone, but in their integration into a short sequence-matched modular peptide system. Here, we compared two sequence-matched peptides, ug51 and ug52, differing only by *N*-terminal acetylation. We examined whether this single terminal modification alters spontaneous degradation, fibril formation, MMP-7-mediated processing, and osteoblast-associated biological responses. We hypothesized that *N*-terminal acetylation would reduce the susceptibility of the modular peptide to spontaneous cleavage and favor the formation of more persistent supramolecular assemblies while preserving the accessibility of the PLGL linker to enzymatic processing. Thus, this work directly tests whether N-terminal acetylation can shift this particular modular system from spontaneous degradation toward a more stable, fibril-forming, protease-processable state.

## Materials and methods

### Peptide synthesis and purification

Peptides were synthesized by Fmoc solid-phase peptide synthesis using an automated microwave-assisted Liberty Blue peptide synthesizer (CEM Corporation) operating under a nitrogen atmosphere. All peptides were synthesized on a 0.25 mmol scale using Rink Amide ProTide resin (CEM Corporation; loading 0.59 mmol/g; 0.424 g). Standard couplings were performed using 4 equiv. of Fmoc-protected amino acid, 4 equiv. of N,N′-diisopropylcarbodiimide (DIC), and 4 equiv. of Oxyma Pure relative to resin loading. The corresponding stock solutions were 0.2 M Fmoc-amino acids, 0.5 M DIC, and 1.0 M Oxyma Pure in DMF. Fmoc deprotection was carried out with 20% piperidine in DMF. Standard coupling was performed under microwave irradiation at 75 °C for 15 s at 175 W, followed by 90 °C for 230 s at 30 W, with intermittent nitrogen flow of 2 s on/3 s off. Fmoc deprotection was performed at 70 °C for 35 s at 150 W, followed by 89 °C for 90 s at 30 W. After each coupling and deprotection step, the resin was washed four times with DMF. Single coupling was used as the standard procedure, while double coupling was applied where required to improve coupling efficiency. For N-terminally acetylated peptides, N-terminal acetylation was performed on resin prior to cleavage by treating the peptidyl resin overnight with 2.75 mL of N-acetylimidazole solution in DMF (1 g/10 mL; 10 equiv. relative to resin loading). After acetylation, the resin was washed thoroughly with DMF. After completion of synthesis, all peptidyl resins were additionally washed four times with DMF and three times with methanol. Peptides were cleaved from the resin with simultaneous side-chain deprotection using a cleavage cocktail composed of TFA/phenol/water/EDT/TIPS at 86.5:5:5:2.5:1 (v:v:v:v:v), using 10 mL of cleavage mixture per 1 g of resin. The suspension was shaken for 3 h at room temperature and filtered under reduced pressure. Crude peptides were precipitated with cold diethyl ether and centrifuged for 15 min at 4 °C and 4000 rpm. The peptide pellet was washed three additional times with diethyl ether, dried from residual ether, dissolved in water, and lyophilized. Crude peptide yields ranged from 76.99 to 96.74%: ug51, 85.93%; ug52, 82.70%; ug54, 76.99%; ug55, 96.74%; and ug1, 95.41%. Crude peptides were purified by semi-preparative RP-HPLC using a Jupiter Proteo C8 column (Phenomenex; 4 µm, 90 Å, 21.2 × 250 mm) with UV detection at 223 nm. The mobile phase consisted of eluent A (0.1 M acetic acid and 0.1 M ammonium acetate in water) and eluent B (65% acetonitrile in 0.1 M acetic acid and 0.1 M ammonium acetate in water), applied as a linear gradient at 1.0 mL/min. Analytical RP-HPLC was performed on a Nexera X2 system (Shimadzu) equipped with a Kinetex C8 column (Phenomenex; 2.6 µm, 100 Å, 2.1 × 100 mm), using eluent A (0.1% TFA in water) and eluent B (80% acetonitrile in 0.1% TFA in water) at 0.5 mL/min. The purity of all purified peptides was >95%, determined by analytical RP-HPLC.

Peptide identity and molecular mass were confirmed by MALDI-TOF mass spectrometry (Bruker Daltonics).

### Stability studies

The stability of the peptides was assessed under aqueous conditions and in cell culture medium. The peptide content was determined by HPLC equipped with an ELSD LTII light scattering detector (Shimadzu) and a Kromasil C8 analytical column (Nouryon; 5 µm, 100 Å, 4.6 mm × 250 mm). The values were normalized to the initial measurement (*t* = 0 h, 100%).

*Stability in water*: Peptide solutions (100 µM) were incubated in water at 37 °C with continuous orbital shaking (300 rpm) for 0, 1, 2, 3, 6, and 24 h. Each time point was analyzed in triplicate.

*Stability in cell culture medium*: Peptide solutions (100 µM) were incubated in DMEM/F-12 medium supplemented with 10% FBS at 37 °C, with orbital shaking (300 rpm) for 0, 1, 2, 3, 6, and 24 h. Following incubation, samples were frozen at −90 °C, thawed on ice for 1 h, and mixed with 1 mL ethanol. Samples were vortexed, centrifuged (18,000 rpm, 20 min, 4 °C), and the supernatant was evaporated in a SpeedVac vacuum concentrator. Residues were reconstituted in 200 µL of phase A (0.1% TFA in water) and 50 µL of phase B (0.1% TFA in 80% acetonitrile/water; v:v) prior to analysis.

*Extended stability assessment of ug51 under selected pH and temperature conditions*: Peptide ug51 was incubated in deionized water (pH 7.2) and in HEPES buffer (pH 6.6) at 37 °C or 40 °C, with continuous mixing in a thermoblock. The pH 7.2 value refers to the measured pH of the peptide solution in deionized water and was not intended to represent a buffered physiological condition. The samples were collected after 0, 2, and 7 days. The degradation products were separated by RP-HPLC using a Nexera X2 system (Shimadzu) and a Kinetex C8 column (Phenomenex; 2.6 µm, 100 Å, 2.1 mm × 100 mm), and identified by MALDI-TOF mass spectrometry (Bruker Daltonics). This extended pH/temperature stability experiment focused on ug51 because it showed pronounced spontaneous degradation in the initial stability assay, while ug52 remained largely intact over 24 h.

### Conformational analysis of peptides by circular dichroism (CD) spectroscopy

Peptide solutions (ug51, ug52) were prepared at a concentration of 1 mg/mL in phosphate-buffered saline (PBS, pH 7.4) and incubated at 37 °C with continuous orbital mixing (300 rpm) in a thermoblock for up to 6 days. The samples were collected at 0, 1, 2, 3, and 6 days. CD spectra were recorded at room temperature in the wavelength range of 190–260 nm using a Jasco J-815 spectropolarimeter. The solvent spectrum was subtracted from each peptide spectrum. Data were expressed as mean residue molar ellipticity (MRME) in units of deg × cm^2^ × dmol^−1^ as a function of wavelength (nm). Measurements were performed in the Circular Dichroism Laboratory of the Faculty of Chemistry of the University of Gdańsk.

### Analysis of peptide fibril aggregation by thioflavin T (ThT) fluorescence

Peptide solutions (ug51, ug52) were prepared at a concentration of 1 mg/mL in phosphate-buffered saline (PBS, pH 7.4) and incubated at 37 °C with continuous orbital mixing (300 rpm) in a thermoblock for up to 6 days. Samples were collected at 0, 1, 2, 3, and 6 days. For each measurement, 10 µL of the peptide solution was mixed with 10 µL of ThT solution (1.5 mM in water) and 90 µL of PBS (pH 7.4). Negative controls consisted of ThT solution mixed with PBS and 10 µL of water. Fluorescence measurements were performed using an Infinite 200 Pro spectrofluorometer (Tecan, Männedorf, Switzerland) on 96-well plates. The excitation wavelength was set at 420 nm and emission spectra were recorded from 455 to 600 nm. Data were collected as fluorescence intensity versus wavelength.

### Imaging and morphometric analysis of peptide ug52 fibrils by transmission electron microscopy (TEM)

Peptide ug52 solutions (1 mg/mL in PBS, pH 7.4) were incubated at 37 °C with continuous orbital mixing (300 rpm) in a thermoblock for up to 6 days. Samples were collected at 0, 1, 2, 3, 4, and 6 days. The fibrils were purified by three successive centrifugation steps (18,000 rpm, 15 min), with the removal of the supernatant and the resuspension of the pellets in deionized water after each step to remove residual buffer. The final concentration of the peptide was adjusted to 0.001% (w:v). For TEM preparation, 5 µL of the fibril suspension was applied to carbon-coated copper grids (400 mesh) and allowed to adsorb for 1 min. Excess liquid was removed, and grids were stained with 2% (v:v) uranyl acetate. Imaging was performed using a Tecnai G2 Spirit BioTWIN transmission electron microscope (FEI Company) operated at 120 kV, with magnifications ranging from 11,500× to 39,000×. Analyses were conducted at the Laboratory of Electron Microscopy, Faculty of Biology, University of Gdańsk. Quantitative TEM morphometry was performed on representative micrographs of ug52 fibrillar assemblies after calibration using the scale bars. Fibril diameters were measured only for clearly resolved, non-overlapping fibril segments. Apparent fibril lengths were measured only when both ends of a fibril or fibrillar segment were visible within the field of view. Measurements affected by strong overlap, poorly defined edges, or image artifacts were excluded. Data are presented as mean ± SD. Measurements were obtained from 16 representative micrographs, including 279 fibril-diameter measurements and 38 measurements of clearly visible fibril segments.

### MMP-7-mediated processing of ug52 fibrillar assemblies

The susceptibility of peptide ug52 fibrils to enzymatic hydrolysis was assessed using matrix metalloproteinase MMP-7 (matrilysin-1; Sigma-Aldrich), selected for its broad substrate specificity and involvement in extracellular matrix remodeling, inflammatory processes, and tissue regeneration. Fibril suspensions (1 mg/mL in PBS, pH 7.4) were incubated with MMP-7 in an enzyme-to-substrate molar ratio of 1:365.6. Incubation was carried out for 72 h at 37 °C with continuous mixing (300 rpm). The samples were collected at 0, 1, 24, and 72 h. Enzymatic activity was stopped by adding trifluoroacetic acid (TFA) to a final concentration of 10% (v:v). Degradation products were analyzed using a Bruker Autoflex III MALDI-TOF mass spectrometer (Bruker Daltonics). The assay was used to confirm the identity of enzymatically generated fragments rather than to determine absolute release kinetics.

### Evaluation of cytotoxicity and proliferation of hFOB 1.19 osteoblast cells

Human fetal osteoblast cells (hFOB) were seeded in 96-well plates at a density of 0.02 × 10^6^ cells per well 1 day prior to the experiment, then incubated for 24 h at 34 °C. Immediately before the experiment, peptide dilutions were prepared in culture medium at concentrations of 100, 50, 25, 12.5, and 6.25 μg/mL. Aliquots (150 μL) of each dilution were added to cell cultures in two independent series, each performed in quadruplicate. After 48 h of incubation at 34 °C, biological assays were performed. Appropriate positive and negative controls were included in all experiments.

*Lactate dehydrogenase (LDH) assay*: Cytotoxicity was evaluated according to ISO 10993-5. Cells were incubated with test peptides for 48 h in 96-well plates. A 1% Triton X-100 solution was used as a positive control. Following incubation, dye solution with catalyst was added to each well immediately before use, and plates were incubated in the dark for 30 min at room temperature. Absorbance was measured at 490 nm with background correction at 690 nm using a microplate reader.

*WST-1 assay*: The effect of peptides on cell metabolic activity was assessed according to ISO 10993-5 using hFOB 1.19 cells. One milliliter of WST-1 reagent solution was pre-warmed in a 34 °C water bath and aliquots were added to each well containing cell cultures. The plates were incubated for 2 h at 34 °C, the reaction was stopped with stop solution, and the absorbance was measured at 450 nm (test) and 620 nm (background) using a microplate reader. Data are presented as mean ± SD. For ug1, ug54, and ug55, WST-1 measurements were performed in four technical replicates, while ug51 and ug52 were analyzed in two technical replicates. The WST-1 results for ug51 and ug52 were descriptively interpreted and supported by independent cell number analysis.

### MTT cytocompatibility assay (L929 and hFOB 1.19)

The cytocompatibility of ug51 and ug52 was evaluated in L929 fibroblasts and hFOB 1.19 osteoblasts using the MTT assay (ISO 10993-5 framework). Cells were seeded in 96-well plates (100 µL/well) at 2 × 10^4^ cells/well (L929) or 4 × 10^4^ cells/well (hFOB 1.19) and allowed to adhere overnight. L929 cells were cultured at 37 °C, while hFOB 1.19 cells were maintained at 33 °C, both in a humidified 5% CO_2_ atmosphere. Cells were exposed for 24 h to twofold serial dilutions of ug51 (0.78–1600 µg/mL) or ug52 (0.49–1000 µg/mL). Untreated cells served as the non-treated control (NTC), while 10% (v/v) DMSO was used as a treated control (TC). After exposure, MTT was added to a final concentration of 0.5 mg/mL and incubated for 4 h. The supernatant was removed, the obtained formazan crystals were dissolved in DMSO, and absorbance was read at 570 nm with background correction at 630–690 nm. Cell metabolic activity was normalized to the NTC and expressed as a percentage. A 70% viability threshold was applied. Peptide concentrations yielding values ≥70% were considered cytocompatible. All conditions were tested in ≥6 replicates.

### CyQUANT-based quantification of hFOB 1.19 cell proliferation

The proliferative effects of ug51 and ug52 (each at 50 µg/mL) on osteoblast hFOB 1.19 were evaluated using the CyQUANT™ cell proliferation assay (Invitrogen, Thermo Fisher Scientific), as previously described (Urbaniak et al. 2025). Cells cultured under identical conditions without peptides served as the untreated control. The cell number was quantified after 1, 2, 3, and 5 days of incubation. At each time point, the samples were washed with PBS and stored at −80 °C. Before DNA quantification, the samples were thawed at room temperature and lysed for 5 min in lysis buffer containing CyQUANT-GR dye, which selectively stains cellular DNA. Fluorescence was measured at 480 nm excitation and 520 nm emission using a SpectraMax® i3x Multi-Mode Microplate Reader (Molecular Devices). Standard curves were generated using serial dilutions of cell suspensions with known cell numbers and were used to convert fluorescence signals into cell counts.

### Cell migration and wound-closure response evaluated using a scratch/wound-healing assay

Cell migration/regeneration capacity in response to ug51 and ug52 was evaluated using an in vitro scratch (wound-healing) assay as previously described (Szwed-Georgiou et al. 2026). Briefly, hFOB 1.19 osteoblasts were plated in 12-well plates at a density sufficient to obtain a confluent monolayer in 24 h (0.5 × 10^6^ cells per well in 1 mL). After monolayer formation, a reproducible “wound” was generated by dragging a sterile pipette tip across the cell layer in two orthogonal directions, producing a cross-shaped scratch. The wells were gently rinsed with PBS to remove detached cells, and fresh medium containing ug51 or ug52 was added at final concentrations of 50, 400, or 800 µg/mL; untreated wells (medium only) served as control. Phase-contrast images of the same wound regions were collected immediately after scratching (0 h) and after 24, 48, and 72 h of incubation, using an inverted microscope. For each well, four non-overlapping fields along the wound margins were documented. Wound closure was quantified by image analysis (ImageJ) as the reduction in scratch area over time and reported as the percentage of wound coverage relative to the initial wound area (0 h), allowing normalization between wells and conditions.

### Chemotaxis assay

Chemotactic migration was assessed using µ-Slide Chemotaxis (ibiTreat; ibidi), which contains three independent cell reservoirs, each connected to two adjacent chambers, allowing the establishment of defined soluble-factor conditions. Cells were seeded in each reservoir according to the manufacturer’s format and allowed to settle. Subsequently, the adjacent chambers were filled to generate the following experimental configurations: (i) peptide-containing medium in the left chamber and culture medium alone in the right chamber (directional gradient condition); (ii) culture medium in both chambers (random migration control); and (iii) peptide-containing medium in both chambers (uniform exposure condition to evaluate non-directional effects).

Cells were imaged by bright-field microscopy 1 h after loading the peptide-containing medium and/or culture medium. After 24 h of incubation, cells were fixed with 3.7% paraformaldehyde (PFA, Sigma-Aldrich) and stained with DAPI (Thermo Fisher Scientific) for 15 min (1 µg/mL) and DiOC_6_ (Thermo Fisher Scientific) for 20 min (0.1 µg/mL). Fluorescence imaging was performed using a Leica Mica Microhub microscope. Image analysis was performed using the Leica Application Suite X software LASX_Office_1.4.3_26413 (LAS X, Leica Microsystems).

### Statistical analysis

Data are presented as mean ± SD or mean ± SEM, as indicated in the figure legends. Statistical comparisons were performed using the Mann-Whitney *U* test. Differences were considered statistically significant at *p* < 0.05. The number of replicates is provided in the corresponding methods and the figure legends.

## Results

### Design of peptides

**Fig. 1:**
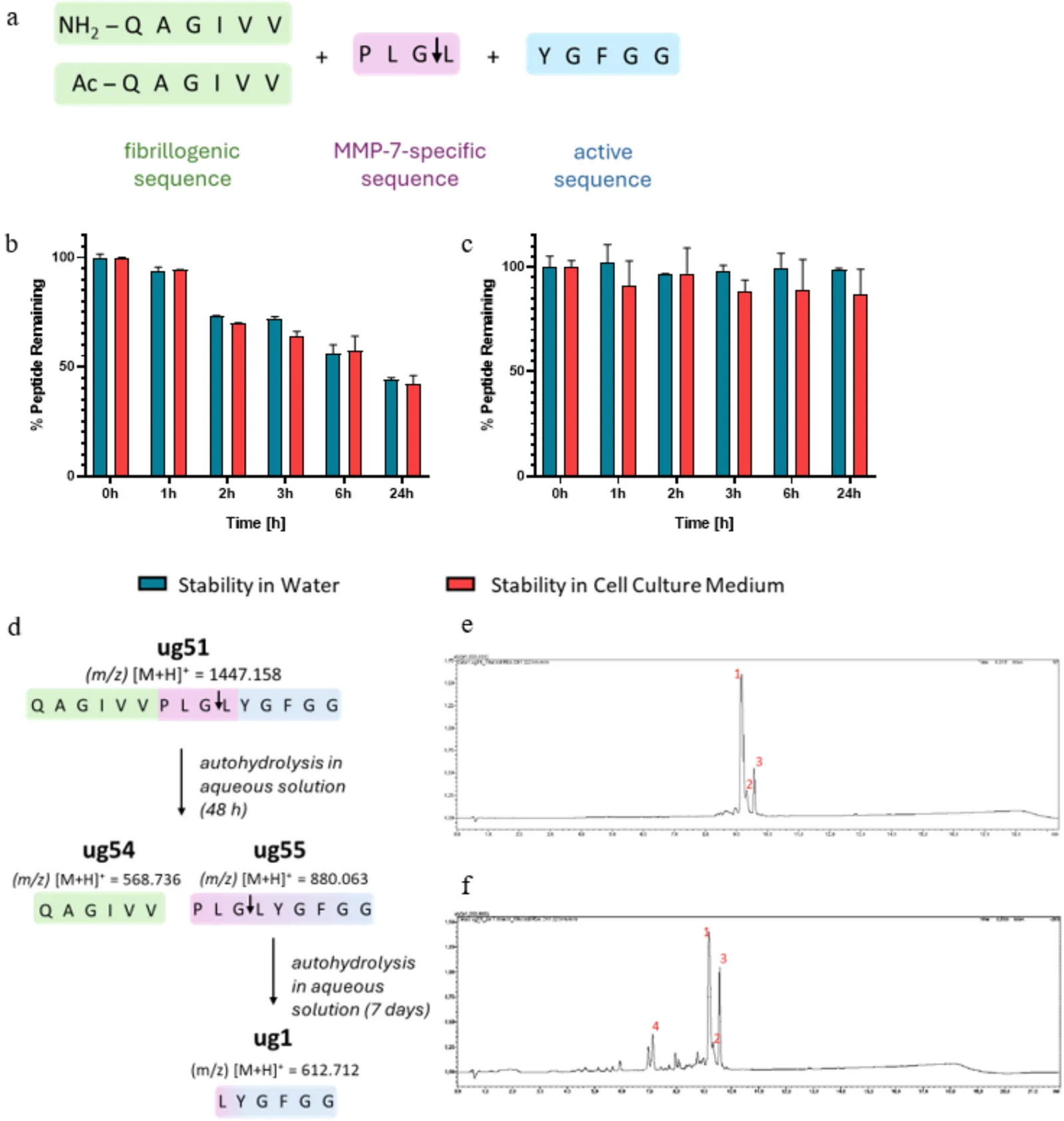
Modular design and degradation analysis of ug peptides. **a** Schematic representation of the peptide design combining structural and bioactive elements. The fibrillogenic sequence QAGIVV (green) promotes self-assembly into β-sheet-rich nanofibers. The MMP-7-sensitive PLGL linker (magenta) contains the proteolytic cleavage site (arrow) recognized by matrix metalloproteinase-7, allowing MMP-7-mediated processing with detectable formation of the OGP-related fragment LYGFGG, which contains the OGP-derived YGFGG motif (blue). Peptides ug51 and ug52 contain the fibrillogenic domain with a free *N*-terminus (NH_2_-QAGIVV) or an acetylated *N*-terminus (Ac-QAGIVV). Stability profiles of peptides in water and cell culture medium: **b** ug51 and **c** ug52. Data represent mean values ± standard deviation (SD) from three independent measurements. **d** Amino acid sequence of ug51 (QAGIVVPLGLYGFGG) with fragments generated during spontaneous degradation detected by MALDI-MS. After 48 h, the fragments QAGIVV ([M + H]^+^ m/z = 568.736) and PLGLYGFGG ([M + H]^+^ m/z = 880.063) were observed. After 7 days, an additional fragment LYGFGG was detected ([M + H]^+^ m/z = 612.712; [M + K]^+^ m/z = 650.340). Representative HPLC chromatograms illustrating the degradation of ug51 after 48 h (**e**) and 7 days (**f**). The peaks correspond to (1) *t*_r_ = 9.17 min, [M + H]^+^ m/z = 1447.158 (ug51); (2) *t*_r_ = 9.32 min, [M + H]^+^ m/z = 880.063; (3) *t*_r_ = 9.56 min, [M + H]^+^ m/z = 568.736; and (4) *t*_r_ = 7.12 min, [M + H]^+^ m/z = 612.712

The peptides were designed using a modular strategy that integrates self-assembly, enzyme responsiveness, and bioactivity within a single sequence (Online Resource 1, Table S1). Ug51 and ug52 contain the *N*-terminal QAGIVV segment (Fig. 1a, green), which was introduced to promote self-assembly in peptide nanofibril scaffolds (Sawicka et al. 2021). This segment is followed by the PLGL linker (Fig. 1a, magenta), which provides an MMP-7-sensitive cleavage site, and by the *C*-terminal YGFGG sequence shown in blue in Fig. 1a. Thus, the expected OGP-related LYGFGG fragment comprises the terminal leucine of the PLGL linker and the C-terminal YGFGG sequence (Pigossi et al. 2016). The two full-length peptides differ only in the presence of *N*-terminal acetylation in ug52. This modification was introduced to modulate stability and self-assembly while preserving the MMP-7 cleavage site and the potentially bioactive OGP-related fragment.

### Stability of peptides ug51 and ug52 in water and cell culture medium

Peptides ug51 and ug52 were obtained by synthesis of solid-phase Fmoc peptides and purified by RP-HPLC to >95% purity, as confirmed by analytical chromatograms (Online Resource 1, Figs. S1 and S3). Their identities and molecular masses were verified by MALDI-TOF MS, with the observed m/z values corresponding to the expected molecular ions or adducts (Online Resource 1: Figs. S2 and S4, Table S1). To assess whether *N*-terminal acetylation affects peptide stability under aqueous and biologically relevant conditions, ug51 and ug52 were incubated in water and in DMEM/F-12 medium supplemented with 10% FBS at 37 °C for 24 h.

HPLC analysis showed that *N*-terminal acetylation was associated with markedly improved apparent stability of ug52 under the tested conditions. After 24 h, the non-acetylated peptide ug51 retained only 44.0% ± 1.1% of its initial amount in water and 42.1% ± 3.9% in cell culture medium. In contrast, the acetylated analog ug52 remained largely intact, with 98.7% ± 0.7% remaining in water and 87.2% ± 11.7% in cell culture medium. Data are presented as mean ± SD. The time-course profiles confirmed this difference between the two peptides (Fig. 1b and c). Ug51 showed a progressive decrease from the beginning of incubation, with approximately half of the peptide degraded within the first 6 h in both environments. By contrast, ug52 showed only minor loss during the same period, indicating that the *N*-terminal acetyl group was associated with substantially reduced loss of the parent peptide, potentially reflecting both local chemical and assembly-related effects not only in water but also in the presence of serum-containing culture medium.

Chromatographic analysis of ug51 degradation revealed the formation of two major fragments, consistent with spontaneous cleavage at the Val^6^-Pro^7^ peptide bond (Fig. 1d and e). After 48 h of incubation in water at 37 °C, the signal of the parent peptide decreased, and two degradation products were detected by RP-HPLC and identified by MALDI-TOF MS as fragments corresponding to the QAGIVV and PLGLYGFGG regions. Prolonged incubation for 7 days resulted in the appearance of an additional product assigned to LYGFGG, corresponding to ug1, the bioactive *C*-terminal fragment (Fig. 1f; Online Resource 1: Table S2). This fragmentation pattern indicates that ug51 primarily undergoes autohydrolysis at the Val^6^-Pro^7^ site, followed by slower secondary cleavage within the PLGLYGFGG fragment.

The observed susceptibility of the Val-Pro bond is consistent with previous reports showing that peptide bonds adjacent to proline can be prone to non-enzymatic cleavage because of the distinctive conformational and electronic properties of proline-containing motifs (Vanhoof et al. 1995; Wedemeyer et al. 2002; Butré et al. 2015). In contrast, the absence of analogous degradation products for ug52 under the same conditions suggests that *N*-terminal acetylation suppresses spontaneous autohydrolysis and stabilizes the full-length peptide. To further evaluate whether the degradation products of ug51 retain biological relevance, the corresponding fragments QAGIVV-NH_2_ (ug54) and PLGLYGFGG-NH_2_ (ug55) were synthesized and characterized by HPLC and MALDI-TOF MS prior to biological testing (Online Resource 1: Table S2).

### Identification and synthesis of degradation products and stability of ug51 under different pH and temperature conditions

HPLC and MS analysis of degraded ug51 samples identified three main cleavage products ug54, ug55 and ug1, respectively corresponding to the N-terminal fibrillogenic fragment, a fragment containing the C-terminal bioactive sequence and the protease recognition sequence, and a fragment containing the active sequence; the first two of these peptides were synthesized by standard SPPS with yields >75%, purified to >95% by HPLC, and their identities confirmed by MALDI-TOF MS (Table S2). Under the exploratory conditions tested, ug51 degradation was more pronounced in HEPES buffer at pH 6.6 than in unbuffered deionized water with an initial measured pH of 7.2, particularly at 40 °C. Because pH and buffer composition were not varied independently, this comparison does not establish a pH-dependent stability profile; detailed experimental data and extended analysis are provided in Online Resource 1.

### Secondary structure analysis of ug51 and ug52 by circular dichroism (CD)

**Fig. 2:**
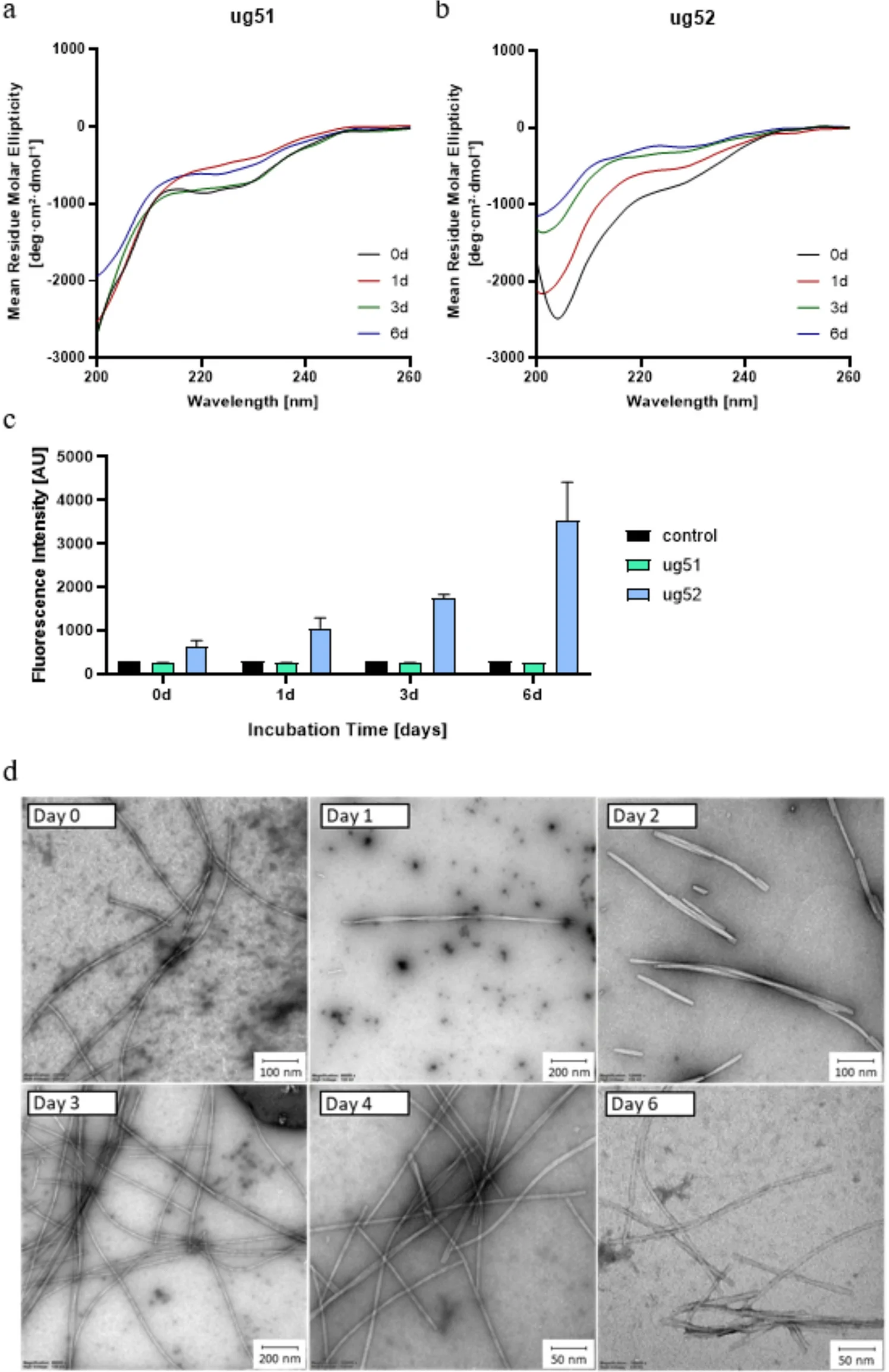
Structural evolution and fibrillogenesis of ug peptides. Circular dichroism (CD) spectra of ug51 (**a**) and ug52 (**b**) recorded after 0, 1, 3, and 6 days of incubation in PBS at 37 °C under continuous agitation. **c** Thioflavin T (ThT) fluorescence assay showing the fluorescence intensity of the peptide-ThT complexes for ug51 and ug52 as a function of the incubation time relative to the PBS control. Data represent mean values ± standard deviation (SD) from three independent measurements. **d** Transmission electron microscopy (TEM) images illustrating the morphological evolution of ug52 fibrils after 0, 1, 2, 3, 4, and 6 days of incubation in PBS. Progressive formation of elongated nanofibrillar structures is observed over time

To investigate fibrillization-related conformational changes, the secondary structure of ug51 and ug52 was monitored by far-UV CD spectroscopy during 6 days of incubation in PBS (pH 7.4) at 37 °C under continuous agitation (Fig. 2a and b). Ug51 showed a spectrum typical of a predominantly random coil structure, with a pronounced negative band at ~200 nm. The spectra recorded at 0, 1, 3, and 6 days were nearly superimposable, indicating that there was no major conformational rearrangement or detectable fibrillization under the tested conditions.

In contrast, ug52 showed a distinct initial spectrum, with negative minima at ~208 and ~222 nm, suggesting a partial α-helical contribution together with a disordered structure. During incubation, the ~208 nm minimum shifted to ~200 nm and ellipticity at both ~208 and ~222 nm decreased. On days 3 and 6, the spectra were dominated by a single minimum near ~200 nm, indicating a predominance of disordered structure in the soluble fraction. These time-dependent spectral changes suggest structural reorganization of ug52, likely associated with peptide aggregation and/or fibril formation, which can reduce the amount of soluble peptide detectable by CD.

### Thioflavin T (ThT) fluorescence assay for ug51 and ug52

The formation of β-sheet-rich fibrillar assemblies by ug51 and ug52 was evaluated using the fluorescence assay of thioflavin T (ThT) during 6 days of incubation in PBS (pH 7.4) at 37 °C under continuous agitation (Fig. 2c). ThT fluorescence increases with binding to cross-β structures and is commonly used as an indirect indicator of amyloid-like fibril formation (Nilsson 2004). Ug51 showed fluorescence values close to baseline throughout the incubation period, with no significant differences compared to the PBS control, indicating that there was no detectable ThT-positive fibril formation under the tested conditions (Fig. 2c).

In contrast, ug52 exhibited a time-dependent increase in ThT fluorescence (Fig. 2c). The signal increased modestly after 1 day, further increased on day 3, and reached its highest intensity on day 6, with statistically significant differences from the control from day 3 onward. This kinetic profile indicates that ug52 undergoes progressive self-assembly into β-sheet-rich amyloid-like fibrillar structures.

### TEM analysis of fibril morphology and morphometry of ug52

Transmission electron microscopy (TEM) was used to follow the morphological evolution of the ug52 assemblies during incubation in PBS (pH 7.4) at 37 °C for up to 6 days (Fig. 2d). On day 0, mainly amorphous aggregates and short protofibrillar structures were observed. After 1 day, thin fibrils appeared, and on day 2 they became longer, more abundant, and partially laterally associated. From day 3 onward, ug52 formed well-defined elongated fibrils and extended fibrillar networks. On day 4, the structures matured further into entangled fibrillar meshes with crossover points and thicker bundles, indicating progressive fibril growth and lateral association. Quantitative morphometric analysis of representative TEM micrographs showed that ug52 fibrillar assemblies had an average apparent diameter of 9.4 ± 2.9 nm, with values ranging from 5.1 to 18.1 nm. For clearly resolved fibrils or fibrillar segments with both ends visible in the field of view, the apparent visible length was 208.8 ± 141.4 nm, with values ranging from 61.5 to 569.6 nm. Because many fibrils overlapped, formed entangled networks, or extended beyond the field of view, the length values should be interpreted as visible segment lengths rather than as a complete fibril length distribution.

### MMP-7-mediated processing of ug52 fibrillar assemblies

To verify whether ug52 fibrils remain susceptible to enzymatic processing, preformed fibrils were incubated with MMP-7 for up to 72 h at 37 °C under continuous agitation and analyzed by MALDI-TOF MS at 0, 1, 24, and 72 h. At the initial time point, the dominant signal corresponded to intact ug52 ([M + Na]^+^ = 1511.092). After 1 h of incubation, new peaks appeared at m/z 612.526 [M + H]^+^ and 634.489 [M + Na]^+^, corresponding to the OGP-related fragment ug1, while the parent signal ug52 was still detectable. After 24 h, the ug52 peak was no longer observed and additional fragments containing the fibrillogenic QAGIVV segment and the MMP-7 recognition sequence were detected, including QAGIVVPLG at m/z 917.524 [M + Na]^+^. This fragmentation pattern supports cleavage within the PLGL linker, between Gly and Leu residues, consistent with the substrate preferences reported by MMP-7 (Turk et al. 2001; Eckhard et al. 2016). After 72 h, only cleavage-product signals were detected under the MALDI-TOF MS conditions used, supporting the MMP-7-mediated processing of ug52 fibrils and detectable release of ug1. The corresponding MALDI-TOF MS spectra are provided in Online Resource 1, Fig. S5.

### Cytotoxicity of ug peptides in hFOB 1.19 cells

**Fig. 3:**
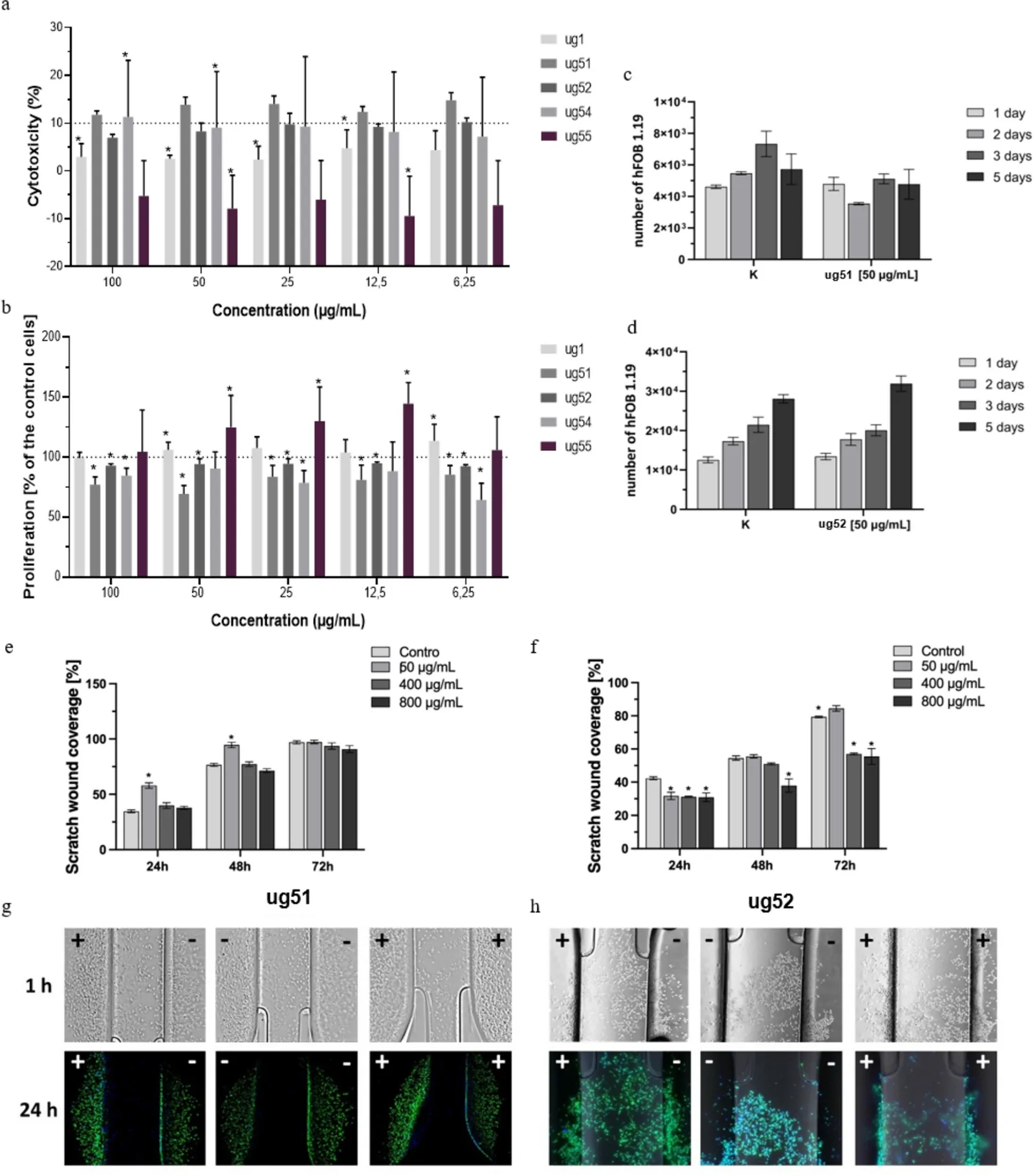
Biological evaluation of ug peptides in hFOB 1.19 osteoblasts. **a** Cytotoxicity of peptides ug1, ug51, ug52, ug54, and ug55 determined using the LDH assay after 48 h of incubation. **b** Effect of peptides ug1, ug51, ug52, ug54, and ug55 on osteoblast metabolic activity measured by the WST-1 assay after 48 h. Data are expressed as percentage of the untreated control (100%). Ug51, ug52, and ug54 showed WST-1 metabolic/proliferation-associated activity comparable to or below the control, whereas ug1 induced a modest increase and ug55 produced the largest increase in the WST-1 signal (up to ~45% above control). Proliferation of hFOB 1.19 osteoblasts cultured with ug51 (**c**) or ug52 (**d**) assessed by the CyQUANT DNA quantification assay. Cells were incubated with peptides at 50 µg/mL, and cell number was measured after 1, 2, 3, and 5 days of culture. K corresponds to untreated cells (control). Migration of hFOB 1.19 osteoblasts evaluated using the scratch wound-healing assay after treatment with ug51 (**e**) or ug52 (**f**) at 50, 400, or 800 µg/mL. Wound closure was quantified at 24, 48, and 72 h and expressed as percentage wound coverage relative to the initial scratch area. Chemotactic response of osteoblasts toward ug51 (**g**) and ug52 (**h**) evaluated using the ibidi µ-Slide Chemotaxis system. Representative images were acquired 1 h after cell loading (bright-field) and after 24 h (fluorescence). Peptides were tested at 50 µg/mL under three chamber configurations: (+/−) gradient (peptide-containing medium vs culture medium), (−/−) medium-only control, and (+/+) uniform peptide exposure. After 24 h, cells were fixed with 3.7% PFA and stained with DiOC_6_ (green; cytoplasmic/membrane labeling) and DAPI (blue; nuclei). Fluorescence images were acquired using a Leica Mica Microhub microscope. Statistical significance was determined using the Mann-Whitney *U* test (*p < 0.05). Data are presented as mean ± SD

To evaluate peptide cytocompatibility and assess potential cytotoxic effects on osteoblasts, hFOB 1.19 cells were analyzed using the LDH assay after 48 h of incubation with ug1, ug51, ug52, ug54, and ug55 at concentrations of 6.25 to 100 µg/mL (Fig. 3a). Across all concentrations tested, none of the peptides exceeded the cytotoxicity threshold (>30% cytotoxicity, corresponding to <70% cell viability). Mean cytotoxicity values remained low and did not differ significantly from the untreated control, indicating no detectable membrane-damage-associated cytotoxicity under these conditions. Additional MTT analysis supported cytocompatibility within the concentration ranges used for the main biological assays, although reduced metabolic activity was observed at the highest tested concentrations in a cell-type-dependent manner (Online Resource 1, Fig. S7).

#### Effect of ug peptides on metabolic/proliferation-associated activity of hFOB 1.19

The WST-1 assay was used to evaluate the effects of ug1, ug51, ug52, ug54, and ug55 on the metabolic activity of hFOB 1.19 cells after 48 h of incubation (Fig. 3b). Peptides ug51, ug52, and ug54 did not significantly affect metabolic activity, with average WST-1 values consistently below or near 100% of control levels. In contrast, ug1 produced a modest yet consistent increase in the WST-1 signal (5–15%). Notably, ug55 produced the largest increase in the WST-1 signal, reaching 20–45% above the control at 12.5–50 µg/mL, with a peak at 12.5 µg/mL. This increase was statistically significant compared with the control (*p* < 0.05) at concentrations of 12.5 and 25 µg/mL.

#### Proliferative response of hFOB 1.19 osteoblasts to ug51 and ug52

To further characterize the effects of the most relevant peptides on osteoblast growth and to directly quantify changes in cell number independent of metabolic activity, the proliferation of hFOB 1.19 cells exposed to ug51 and ug52 was assessed using a CyQUANT DNA quantification assay. This demonstrated a time-dependent increase in hFOB 1.19 osteoblast numbers throughout the culture period (Fig. 3c and d). Exposure to ug51 at 50 µg/mL resulted in cell numbers that remained comparable to the untreated control at all evaluated time points, indicating no effect on osteoblast proliferation under these conditions. In contrast, treatment with ug52 at the same concentration was associated with a more pronounced increase in cell numbers at later stages of culture, suggesting a proliferative trend relative to the control. In general, the data indicate that both peptides were compatible with osteoblast growth, while ug52 exhibited a stronger capacity to support cell expansion during prolonged incubation.

#### Effects of ug51 and ug52 on the migration of hFOB 1.19 osteoblasts in the scratch wound-healing assay

To evaluate whether ug51 and ug52 affect osteoblast motility, migration of hFOB 1.19 cells was analyzed using a scratch wound-healing assay. Wound closure increased with time in all groups, indicating progressive cell migration to the scratched area (Fig. 3e and f). For ug51, the lowest concentration tested, namely 50 µg/mL, accelerated early wound coverage compared to the untreated control, particularly at 24 and 48 h (Fig. 3e). Higher concentrations, specifically 400 and 800 µg/mL, did not provide additional benefit and produced closure values comparable to or slightly below the control. By 72 h, wound closure was nearly complete in all groups, suggesting that the effect of ug51 was mainly limited to the early phase of scratch closure.

In contrast, ug52 showed an overall inhibitory trend in wound closure, especially at higher concentrations (Fig. 3f). At 50 µg/mL, wound coverage was comparable to or slightly higher than control at 48–72 h. However, at 400 and 800 µg/mL, ug52 consistently reduced wound closure, with the strongest attenuation observed at 72 h. These results indicate that ug51 and ug52 modulate osteoblast wound-closure dynamics in a concentration-dependent manner.

#### Evaluation of the chemotactic potential of ug51 and ug52 in hFOB 1.19 osteoblasts

To assess peptide-associated directional migration, ug51 and ug52 were tested using the ibidi µ-Slide Chemotaxis system. Representative images show the distribution of hFOB 1.19 cells exposed to ug51 or ug52, each at 50 µg/mL, under gradient (+/−), medium-only control (−/−), and uniform peptide exposure (+/+) conditions (Fig. 3g and h). Bright-field images acquired 1 h after loading confirmed a comparable initial cell location within the central collagen-filled channel.

After 24 h, peptide and configuration-dependent redistribution patterns were observed. In the ug51 medium-only control, cells partially migrated from the central channel into adjacent medium-filled chambers, consistent with spontaneous, non-directional migration. In the presence of ug51, cell accumulation was more pronounced toward the peptide-containing chamber under gradient conditions, while uniform exposure resulted in migration into both chambers without clear directional bias. For ug52, cells remained mostly within the central channel in the medium-only control, while peptide exposure promoted migration out of the channel. Under the ug52 gradient, cells preferentially redistributed toward the peptide-containing chamber, while uniform exposure to ug52 produced a more symmetric migration pattern. These observations suggest peptide-dependent cell redistribution, with ug52 showing a more evident directional component.

## Discussion

### Peptide stability and degradation behavior

Comparative stability analysis demonstrated that *N*-terminal acetylation markedly altered peptide behavior despite the identical core sequence of ug51 and ug52, which differ only in the presence of the *N*-terminal acetyl group in ug52 (Table S1). Both peptides were obtained with high purity and confirmed by RP-HPLC and MALDI-TOF MS prior to stability testing (Online Resource 1, Figs. S1–S4; Online Resource 1, Table S1). Under aqueous and cell-culture conditions, ug51, which has a free *N*-terminus, was chemically labile, whereas the acetylated analog ug52 remained substantially more stable (Fig. 1b and c). This finding indicates that a single terminal modification can strongly influence the balance between peptide degradation and persistence in biologically relevant environments.

**Fig. 4:**
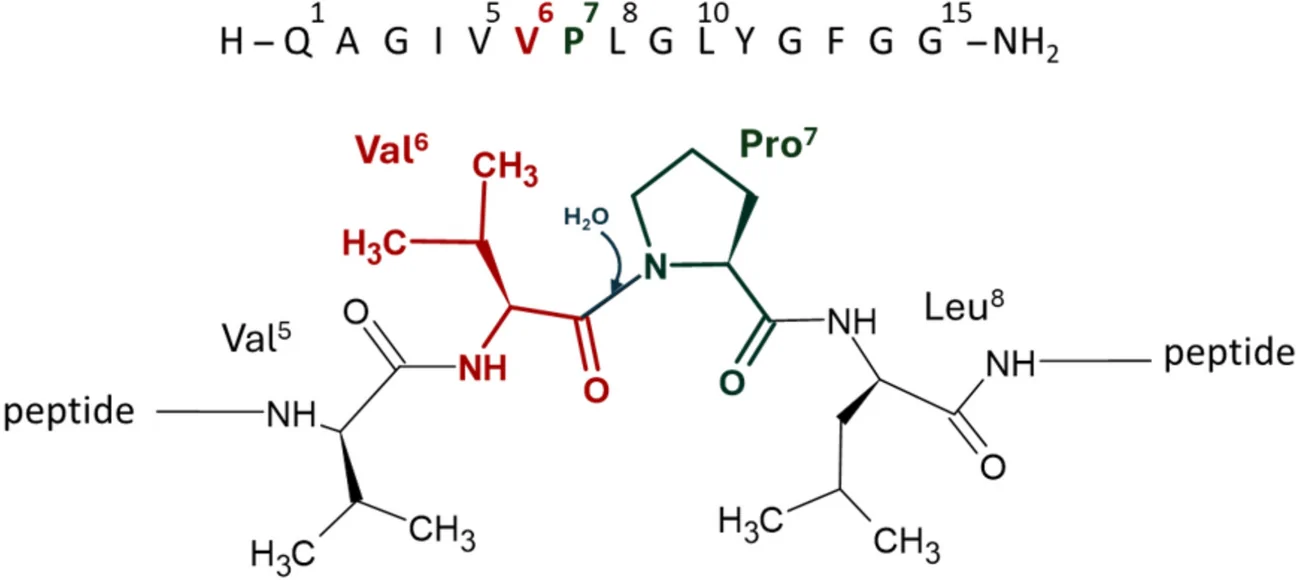
Proposed mechanism of acid-catalyzed cleavage of the Val^6^-Pro^7^ peptide bond in ug51. The nucleophilic attack of water on the carbonyl carbon results in the hydrolysis of the peptide bond and the formation of two peptide fragments

The degradation of ug51 was associated with spontaneous cleavage at the Val^6^-Pro^7^ bond, generating fragments corresponding to the QAGIVV (ug54) and PLGLYGFGG (ug55) regions, followed by slower formation of LYGFGG during prolonged incubation (Fig. 1d–f; Online Resource 1, Table S2). The proposed hydrolytic mechanism involves the cleavage of the peptide bond between Val^6^ and Pro^7^, as illustrated in Fig. 4. Cleavage at peptide bonds preceding proline can occur in a sequence- and condition-dependent manner, particularly under mildly acidic or stress conditions (Landon 1977; Wedemeyer et al. 2002; Butré et al. 2015). The proline cyclic pyrrolidine ring can reduce resonance stabilization of the preceding amide bond and increase susceptibility to protonation and nucleophilic attack, thus facilitating hydrolytic cleavage (Wedemeyer et al. 2002; Umumararungu et al. 2024).

The sequence context may further modulate this process. In ug51, hydrophobic residues surrounding the Val-Pro site can influence local backbone flexibility and solvent exposure, contributing to the susceptibility of this bond to hydrolysis. Similar sequence-dependent effects have been reported for peptides and proteins containing X-Pro or other labile motifs (Vanhoof et al. 1995; Li et al. 2009; Vlasak and Ionescu 2011). The location of the Val^6^-Pro^7^ cleavage site between the fibrillogenic QAGIVV domain and the linker/bioactive region explains the formation of discrete degradation fragments rather than nonspecific fragmentation (Fig. 1d–f). Thus, ug51 can be considered a degradation-prone precursor capable of generating bioactive fragments under aqueous conditions.

Additional stability experiments showed that ug51 degradation was more pronounced under the selected mildly acidic/buffered condition and further enhanced by elevated temperature (Online Resource 1, Fig. S6). The strongest degradation was observed in HEPES buffer at pH 6.6 and 40 °C, suggesting that the incubation environment, including pH, buffer composition, and temperature, may influence spontaneous cleavage. Similar effects of pH and temperature on peptide-bond instability and degradation reactions have been reported previously (Geiger and Clarke 1987). However, this experiment should be interpreted as an exploratory chemical stability assessment under selected conditions rather than as a complete pH-stability profile or as direct evidence of pH-responsive biological activity. Future quantitative studies performed across a broader pH range, including values below the peptide pI and under matched buffer conditions, would be required to define the contribution of pH to ug51 degradation and to determine whether this behavior could be exploited in pH-modulated peptide delivery systems, particularly in environments associated with inflammation, tissue remodeling, or local acidosis (Place et al. 2009; Das et al. 2020). In addition, the exact chemical pathway was not directly determined in the present study; therefore, Fig. 4 should be interpreted as a proposed degradation pathway consistent with the detected fragments rather than as direct mechanistic evidence.

### Effect of *N*-terminal acetylation on peptide stabilization

The markedly higher stability of ug52 highlights the importance of *N*-terminal acetylation as a simple but effective strategy to adjust peptide properties. In the 24-h stability assay, ug52 retained most of its initial amount both in water and in serum-containing cell culture medium, while ug51 underwent substantial degradation over the same period (Fig. 1b and c). *N*-terminal acetylation neutralizes the terminal amino group and can influence local hydrogen bonding, backbone conformation, peptide-peptide interactions, and susceptibility to enzymatic or non-enzymatic degradation (Ngambenjawong et al. 2016; Aksnes et al. 2016). In the present system, acetylation not only improved the chemical stability but also changed the self-assembly behavior of the peptide.

The stabilizing effect of acetylation may result from two complementary mechanisms. First, chemical modification itself may reduce local structural flexibility and alter the exposure of degradation-prone peptide bonds. Second, enhanced self-assembly of ug52 in supramolecular structures may further restrict solvent access to labile regions of the sequence. This interpretation is supported by structural analyses showing that ug52, but not ug51, undergoes a time-dependent assembly into ThT-positive and TEM-visible fibrillar structures (Fig. 2a–d). Therefore, ug52 appears to stabilize both at the molecular level and through higher-order peptide organization.

This distinction is important for the design of peptide nanomaterials. Although ug51 behaves primarily as a labile precursor that spontaneously generates active fragments, ug52 behaves as a more persistent peptide assembly capable of maintaining structural integrity while remaining enzymatically processable. These contrasting behaviors illustrate how terminal modification can redirect the behavior of a modular peptide from degradation-driven activation toward a more stable, nanofibril-forming, protease-processable state.

### Structural transitions and fibrillogenesis

Structural analyses confirmed that ug51 and ug52 follow distinct assembly pathways. CD spectroscopy showed that ug51 maintained a predominantly disordered/random-coil-like conformation during incubation, with no major spectral changes over time (Fig. 2a). This lack of stable secondary structure is consistent with its high chemical lability, as flexible or disordered peptide chains are generally more accessible to hydrolytic cleavage and proteolysis (Deiana et al. 2019; Trivedi and Nagarajaram 2022).

In contrast, ug52 showed a time-dependent structural reorganization. At the initial time point, its CD spectrum contained features consistent with the partial α-helical contribution, while prolonged incubation led to a shift toward a spectrum dominated by a minimum near 200 nm in the soluble fraction (Fig. 2b). *N*-terminal acetylation is known to influence the propensity toward α-helix formation by neutralizing the positive charge at the *N*-terminus of the peptide and reducing the destabilization of the dipole helix (Kang et al. 2012; Moriarty et al. 2013). In the present case, the observed spectral evolution likely reflects early peptide self-association, aggregation, and depletion or reorganization of the soluble peptide fraction rather than a simple transition to a fully disordered state.

This interpretation is supported by ThT fluorescence and TEM imaging. ThT measurements did not show a detectable increase in fluorescence for ug51, while ug52 showed a progressive increase in ThT signal during the 6-day incubation period, consistent with the formation of cross-β-rich fibrillar assemblies (Fig. 2c). The absence of detectable ThT-positive aggregation in ug51 may leave cleavage-prone bonds fully exposed to solvent, facilitating rapid hydrolysis. By contrast, early oligomeric or protofibrillar aggregates formed by ug52 can partially shield susceptible sites such as the Val-Pro region, contributing to its enhanced stability.

TEM imaging further demonstrated that ug52 undergoes time-dependent fibrillogenesis, evolving from amorphous aggregates and short protofibrillar structures into extended nanofibrillar networks (Fig. 2d). Such hierarchical self-assembly, including fibril elongation and lateral association into higher-order bundles, is characteristic of many self-assembling peptide systems and peptide-based nanomaterials (Adler-Abramovich and Gazit 2008; Knowles and Mezzenga 2016). In the present study, *N*-terminal acetylation promoted the formation of stable nanoscale fibrillar assemblies, providing a structural basis for further evaluation of ug52 as a potential scaffold-like peptide-based reservoir that remains susceptible to MMP-7-mediated processing and enables detectable formation of an OGP-related fragment.

### MMP-7-mediated processing of ug52 fibrillar assemblies

A key requirement for enzyme-responsive peptide nanocarriers is their ability to combine structural stability with enzymatic accessibility. The MMP-7 cleavage experiment showed that ug52 fibrils remained susceptible to proteolytic processing despite their supramolecular organization (Online Resource 1, Fig. S5). MALDI-TOF MS detected the OGP-related fragment ug1 after incubation with MMP-7, while the parent ug52 signal was no longer detected after prolonged enzymatic treatment under the analytical conditions applied (Online Resource 1, Fig. S5).

The detection of QAGIVVPLG-containing fragments further supports cleavage within the PLGL linker, consistent with the predicted Gly-Leu processing site in ug52. This cleavage pattern agrees with the known substrate preferences of MMP-7 and other MMPs, including the preference for defined residues around the scissile bond and hydrophobic residues near the cleavage site (Turk et al. 2001; Eckhard et al. 2016). Importantly, fibrillar assembly did not completely shield the enzyme-sensitive linker, indicating that the bioactive *C*-terminal fragment can be released from the assembled peptide material.

Similar behavior has been reported in other fibrillar peptide systems, where flexible or solvent-exposed regions remain accessible to proteolytic enzymes despite the presence of ordered supramolecular structures (Nilsson 2004; Zhang et al. 2009). However, MALDI-TOF MS confirms the identity of the released fragments but does not provide absolute release kinetics. Therefore, the enzymatic experiment should be interpreted as evidence of MMP-7-mediated processing and fragment release rather than as a quantitative release profile.

This enzyme-responsive behavior strengthens the relevance of ug52 for nanobiotechnological applications. The peptide combines three desirable features within a single short sequence: *N*-terminal acetylation-mediated stabilization, self-assembly into β-sheet-rich nanofibrils, and MMP-7-mediated formation of a detectable OGP-related fragment (Fig. 2c and d and Fig. S5). Such a design is particularly relevant for protease-processable peptide assemblies that respond to proteolytic remodeling environments. MMP-7 is involved in extracellular matrix remodeling; therefore, the system may be relevant to protease-rich remodeling microenvironments. However, disease- or tissue-specific responsiveness remains to be demonstrated (Sternlicht and Werb 2001; Sun et al. 2024). Thus, ug52 fibrils may represent an enzyme-responsive peptide reservoir, although further quantitative release and biological validation studies are needed before disease-site specificity can be claimed.

### Cytocompatibility and osteoblast response

The biological assays showed that the investigated peptides and peptide fragments were cytocompatible with hFOB 1.19 osteoblasts within the concentration range tested. LDH analysis did not indicate measurable cytotoxicity for ug1, ug51, ug52, ug54, or ug55 after 48 h of exposure (Fig. 3a). Additional MTT analysis in L929 fibroblasts and hFOB 1.19 osteoblasts further supported the general cytocompatibility of ug51 and ug52, although reduced metabolic activity was observed at the highest concentrations in a cell-type-dependent manner (Online Resource 1, Fig. S7). These findings are consistent with previous studies showing that short peptides composed of natural amino acids generally exhibit low cytotoxicity toward mammalian cells and can be suitable components of biomaterial systems (Schumacher et al. 2010; Collier and Segura 2011).

The WST-1 assay revealed functional differences between the peptide fragments (Fig. 3b). Ug55 produced the strongest increase in osteoblast metabolic/proliferation-associated activity, while ug1 induced a more modest response. This observation is consistent with previous reports linking OGP-derived motifs with stimulation of osteoblast or mesenchymal cell proliferation and bone-related activity (Fei et al. 2010; Pigossi et al. 2016). The activity of ug55 suggests that fragments generated through spontaneous or enzymatic cleavage can contribute to biological effects in situ. In contrast, ug51 and ug52 showed limited effects in the short-term WST-1 assay, indicating that full-length peptides may require degradation, enzyme processing, or longer exposure to reveal stronger biological activity.

Because WST-1 reflects cellular metabolic activity rather than direct cell number, these results were interpreted together with CyQUANT-based DNA quantification. This analysis showed that cultures treated with ug51 remained comparable to controls not treated, while ug52 was associated with a stronger increase in cell number at later times (Fig. 3c and d). This observation suggests that the acetylated self-assembling peptide may support osteoblast growth during prolonged culture. This effect may be related not only to the detectable formation of an OGP-related fragment but also to the presence of peptide assemblies that may alter the local cellular microenvironment. However, further studies using osteogenic differentiation markers, such as ALP activity, mineralization assays, and osteogenic gene expression, are required to confirm a direct pro-osteogenic mechanism.

### Effects on migration-associated responses

Migration-related assays provided additional information on the cellular effects of ug51 and ug52. In the scratch wound-healing assay, ug51 accelerated early wound closure at the lowest concentration tested, particularly at 24 and 48 h, while higher concentrations did not provide any additional benefit (Fig. 3e). This may reflect a concentration-dependent effect of ug51 or its degradation products on the early activity associated with osteoblast migration. By 72 h, wound closure was nearly complete in all groups, suggesting that the effect of ug51 was mainly limited to the early phase of response.

In contrast, ug52 showed an inhibitory trend at higher concentrations, particularly at 400 and 800 µg/mL (Fig. 3f). This may indicate that fibrillar or aggregated peptide structures influence cell movement or alter the physical microenvironment surrounding the cells. Such effects are plausible for self-assembling peptide materials, where the supramolecular organization can affect cell-material interactions, local viscosity, matrix-like properties, or accessibility of bioactive motifs. Therefore, the different scratch assay responses of ug51 and ug52 likely reflect not only the sequence composition but also differences in degradation behavior and supramolecular structure.

The µ-Slide chemotaxis assay further revealed differences in how the two peptides influence osteoblast redistribution (Fig. 3g and h). Peptide ug51 induced a less directional migration pattern, consistent with spontaneous redistribution from the collagen matrix rather than a strong chemotactic signal. In contrast, ug52 promoted preferential cell redistribution to peptide-containing regions under gradient conditions, whereas uniform exposure resulted in a more symmetric distribution (Fig. 3h). This behavior may reflect interactions between peptide assemblies and cell surface receptors or extracellular matrix components. However, because the current assay was based primarily on endpoint imaging, quantitative cell tracking will be needed to confirm a true chemotactic response and distinguish directional migration from general peptide-associated redistribution.

### Design implications and limitations

Together, the results identify *N*-terminal acetylation as an effective terminal modification to tune the behavior of modular self-assembled peptides. Importantly, the conceptual novelty of this study arises from integrating N-terminal acetylation, fibril-forming, MMP-sensitive, and OGP-related elements within a sequence-matched modular peptide pair, thereby allowing the effect of a single terminal modification on stability, assembly, and protease processability to be evaluated directly. In ug51, the absence of acetylation favored chemical lability and spontaneous generation of ug55, a fragment associated with increased osteoblast metabolic/proliferation-associated activity (Figs. 1d–f and 3b; Online Resource 1, Table S2). In ug52, acetylation shifted the system toward enhanced stability and β-sheet-rich nanofibril formation, while preserving susceptibility to MMP-7-mediated processing and allowing detectable release of ug1 (Figs. 1b, c, 2c, d and Online Resource 1, Fig. S5). These contrasting outcomes demonstrate that subtle terminal modifications can be used to tune the stability of the peptide, the supramolecular assembly, and the protease-mediated availability of bioactive fragments.

The study also highlights a broader design principle for enzyme-responsive peptide nanomaterials: the assembled structure must be stable enough to act as a reservoir but not compact enough so that the protease-sensitive linker becomes inaccessible. Peptide ug52 meets this requirement by forming relatively stable fibrillar assemblies that remain susceptible to MMP-7-mediated processing. This balance between apparent stability and enzymatic processability supports the further evaluation of acetylation-tuned peptide nanofibrils as cytocompatible, protease-processable assemblies for bioactive-sequence delivery, although quantitative release studies are required to establish nanocarrier functionality.

Several limitations should be considered. First, MALDI-TOF MS confirmed the identity of the cleavage products but did not provide quantitative release kinetics (Online Resource 1, Fig. S5). Second, although quantitative TEM morphometry was included in the present study, the physicochemical characterization of the fibrillar assemblies remains limited and would benefit from complementary nanoscale characterization in future studies. Third, the WST-1 results reflect metabolic activity and were therefore interpreted together with direct DNA-based cell quantification (Fig. 3b–d). Finally, migration and chemotaxis assays provide early in vitro evidence of peptide-dependent cellular responses, but quantitative cell tracking and osteogenic differentiation endpoints, such as ALP (alkaline phosphatase) activity, matrix mineralization, and osteogenic gene expression, are needed to define the regenerative mechanism more precisely (Fig. 3e–h). Addressing these points in future work will strengthen the translational relevance of this modular peptide platform.

## Conclusions

In general, these findings provide proof-of-concept evidence that *N*-terminal acetylation can modulate the stability, self-assembly, MMP-7-mediated processability, and cell-associated responses of short modular peptides. By comparing two sequence-matched peptides, ug51 and ug52, we showed that a single terminal modification is associated with a shift in peptide behavior from spontaneous autohydrolysis toward more stable nanofibrillar assembly. However, the present two-peptide comparison does not fully separate the direct chemical effect of *N*-terminal acetylation from assembly-mediated stabilization.

The non-acetylated peptide ug51 was chemically labile and underwent spontaneous cleavage in the Val^6^-Pro^7^ region, generating defined fragments, including ug55, which showed pronounced osteoblast metabolic/proliferation-associated activity. In contrast, *N*-terminally acetylated ug52 exhibited markedly improved apparent stability in water and cell culture medium and was assembled into ThT-positive fibrillar assemblies visible by TEM. Importantly, these fibrillar assemblies remained accessible to MMP-7-mediated processing, resulting in detectable formation of the OGP-related fragment ug1.

Together, these findings provide proof-of-concept evidence that structural stabilization and MMP-7-mediated processability can be combined within a single modular peptide sequence. Ug51 may be viewed as a degradation-prone peptide that generates defined fragments under aqueous conditions, while ug52 forms more stable fibrillar assemblies that remain susceptible to enzymatic processing. Although quantitative release kinetics and extended osteogenic validation are still required, these results support the further development of acetylation-tuned peptide nanofibrils as cytocompatible protease-processable assemblies for bioactive-sequence presentation and potential delivery.

In general, the work supports the rational design of enzyme-processable peptide assemblies for biotechnology and regenerative biomaterial applications. Future studies should include quantitative release kinetics, extended osteogenic differentiation assays, and further optimization of terminal modifications to define sequence–structure–function relationships more precisely.

## Supplementary information

Below is the link to the electronic supplementary material.

ESM 1 (PDF 550 KB)

## Data Availability

The data supporting the findings of this study are available in the article and its Electronic Supplementary Material. Additional raw data are available from the corresponding author upon reasonable request.
