# Supplementary material for: N-terminal acetylation modulates peptide stability, fibrillogenesis and MMP-7 processing
Source: Appl Microbiol Biotechnol. 2026 Aug 15;110:343. doi: 10.1007/s00253-026-14007-5 (PMC13755152; doi:10.1007/s00253-026-14007-5)
Supplement: Supplementary file 1 [file 253_2026_14007_MOESM1_ESM.pdf]

Electronic Supplementary Material

Online Resource 1

***Supplementary information for:* Applied Microbiology and Biotechnology**

N-terminal acetylation tunes stability and MMP-7-responsive release in peptide fibrils

Agnieszka Kubiś[1], Justyna Sawicka[1], Marcin Włodarczyk[3], Aleksandra Szwed-Georgiou[3], Mirosława Panasiuk[2], Natalia Karska[1], Beata Gromadzka[2,4], Przemysław Płociński[3], Sylwia Rodziewicz-Motowidło[1#]

[1]Department of Biomedical Chemistry, Faculty of Chemistry, University of Gdańsk, 80-308 Gdańsk, Poland

[2] Department of In Vitro Studies, Institute of Biotechnology and Molecular Medicine, Kampinoska 25, 80-180 Gdańsk, Poland

[3] Department of Immunology and Infectious Biology, Faculty of Biology and Environmental Protection, University of Łódz, Banacha 12/16, 90-237 Łódź, Poland

[4] NanoExpo LTD, Kładki 24, 80-822 Gdańsk, Poland

[#] Corresponding author: S. Rodziewicz-Motowidło; s.rodziewicz-motowidlo@ug.edu.pl

Table S1 Amino acid sequences and analytical characterization of the designed peptides.

Peptide	Amino acid sequence	Retention time (min)	Calculated mass (g/mol)	Observed ion	Observed *(m/z)*
ug51	H–QAGIVVPLGLYGFGG–NH_2	8.66	1446.71	$[M+H]^+$<br>$[M+Na]^+$	1446.763<br>1468.704
ug52	Ac–QAGIVVPLGLYGFGG–NH_2	9.54	1488.75	$[M+Na]^+$<br>$[M+K]^+$	1510.968<br>1526.963

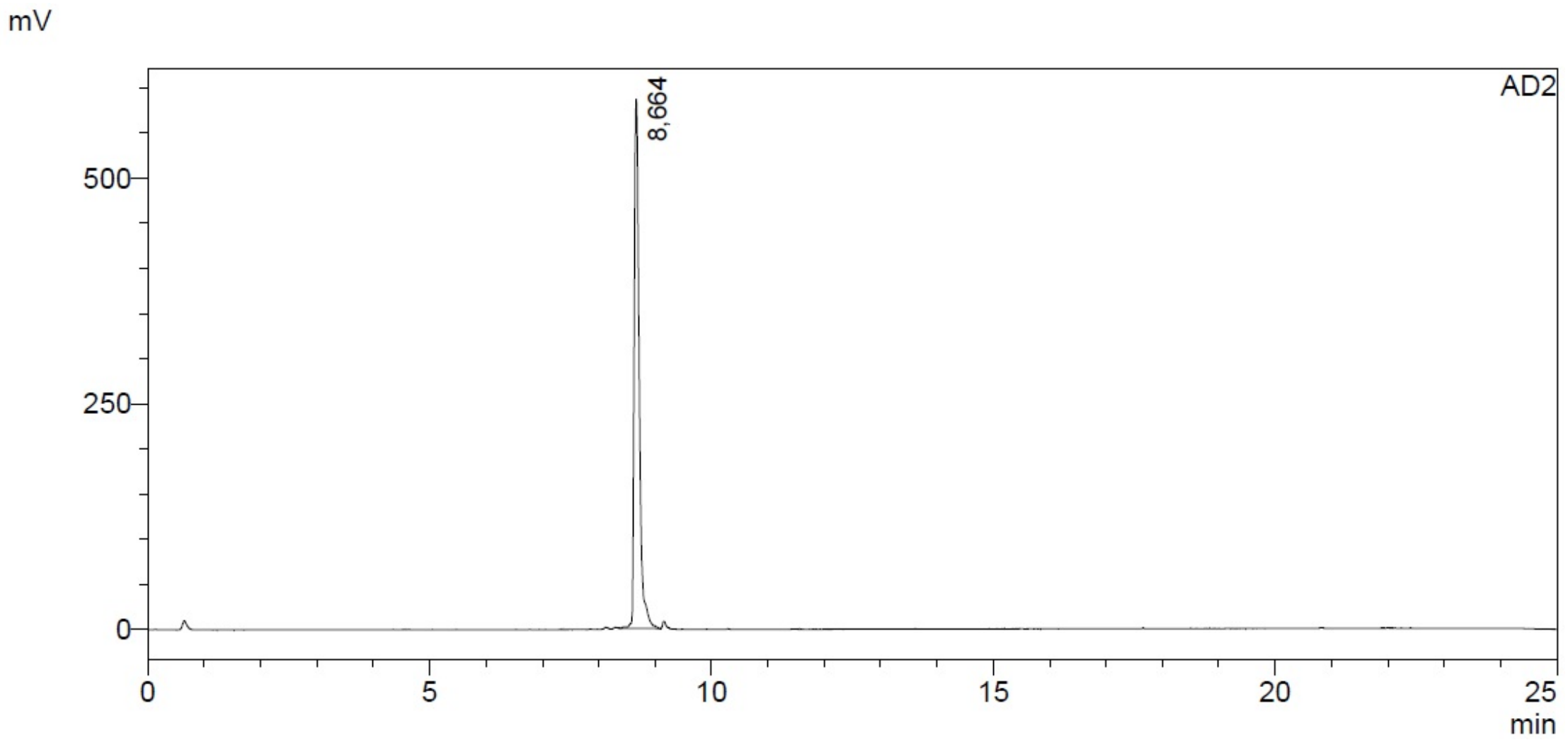

Fig. S1 Chromatogram of peptide ug51 (t_r = 8.66)

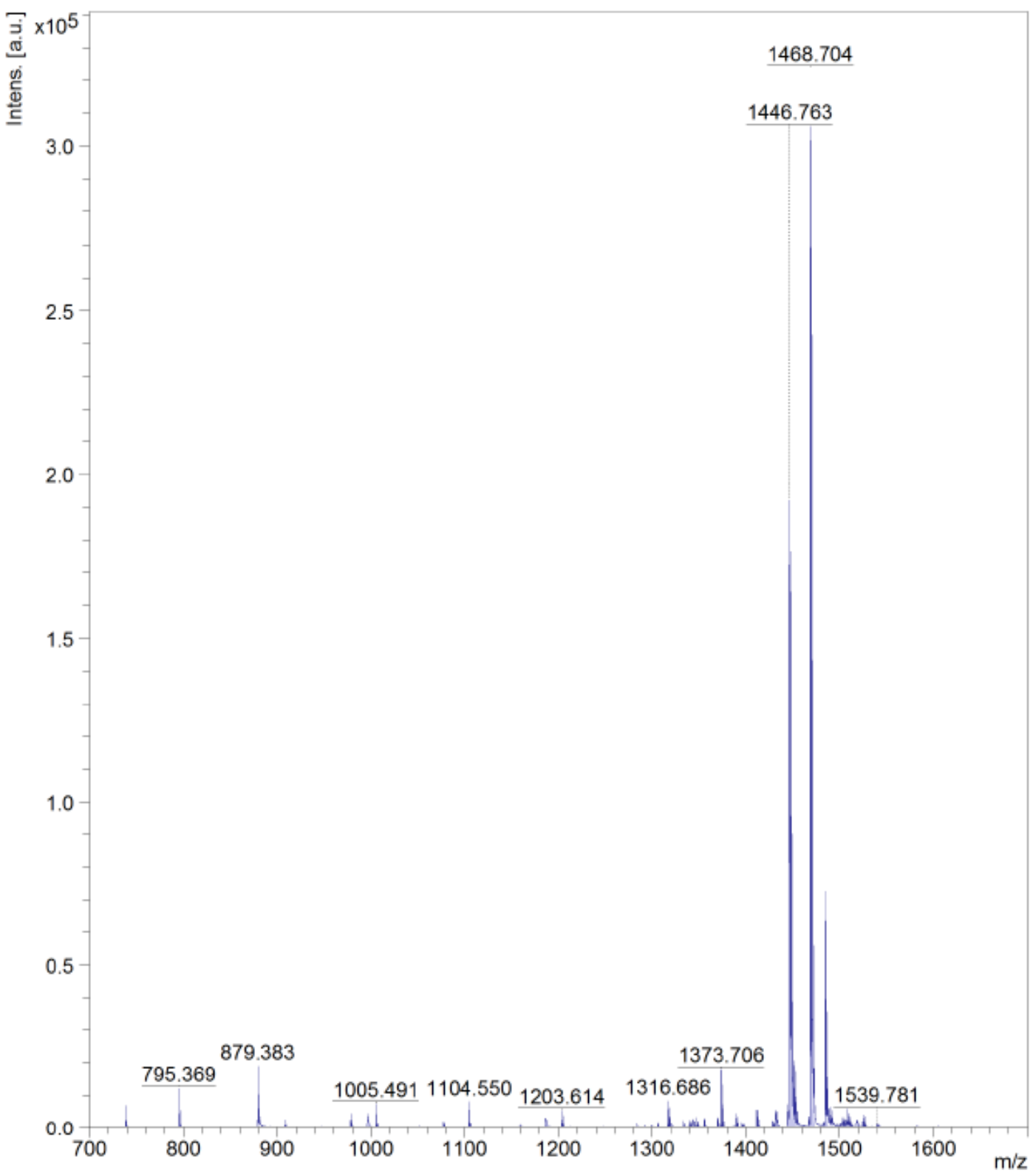

Fig. S2 MALDI–TOF mass spectrum of peptide ug51. Observed peaks: (m/z) $[M+H]^+$ = 1446.763, (m/z) $[M+Na]^+$ = 1468.704

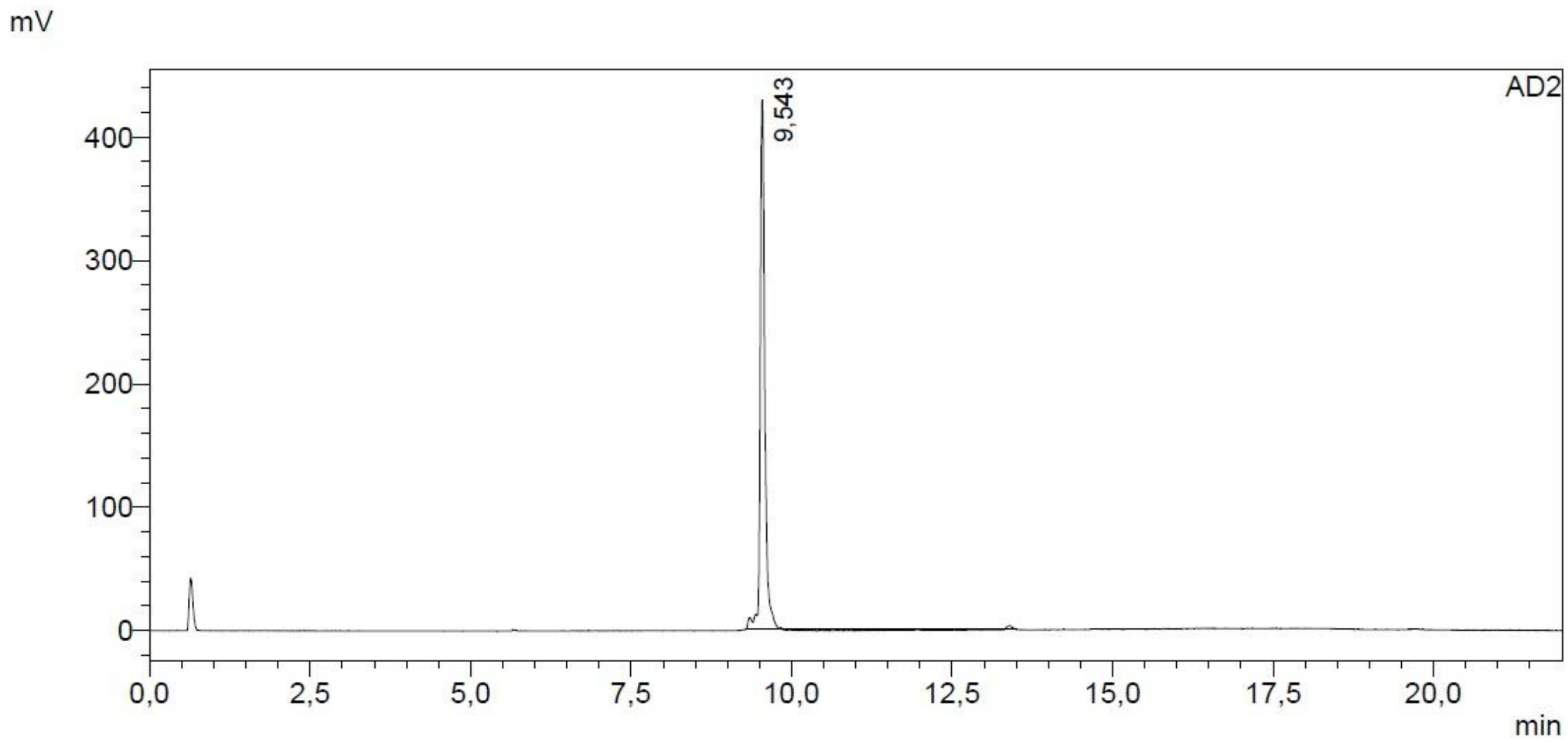

Fig. S3 Chromatogram of peptide ug52 (t_r = 9.54)

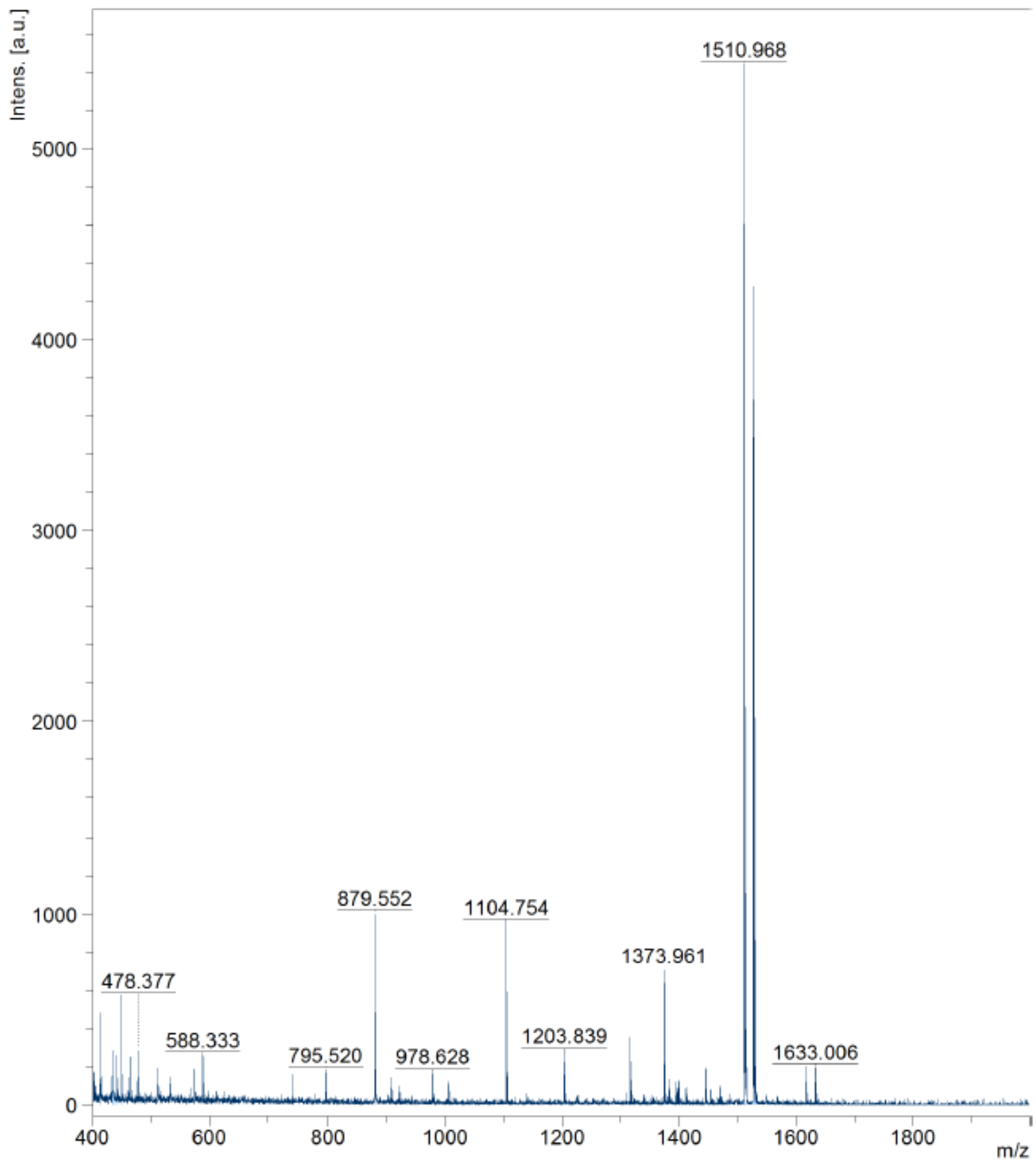

Fig. S4 MALDI–TOF mass spectrum of peptide ug52. Observed peaks: (m/z) $[M+Na]^+$ = 1510.968, (m/z) $[M+K]^+$ = 1526.963

Table S2 Amino acid sequence, retention time and molecular masses of ug1, ug54 and ug55.

**Peptide**	**Amino acid sequence**	**Retention time (min)**	**Calculated mass (g/mol)**	**Observed *(m/z)***
ug1	H–LYGFGG–NH_2	7.12	611.699	611.279
ug54	H–QAGIVV–NH_2	9.32	584.717	584.562
ug55	H–PLGLYGFGG–NH_2	9.56	879.027	878.849

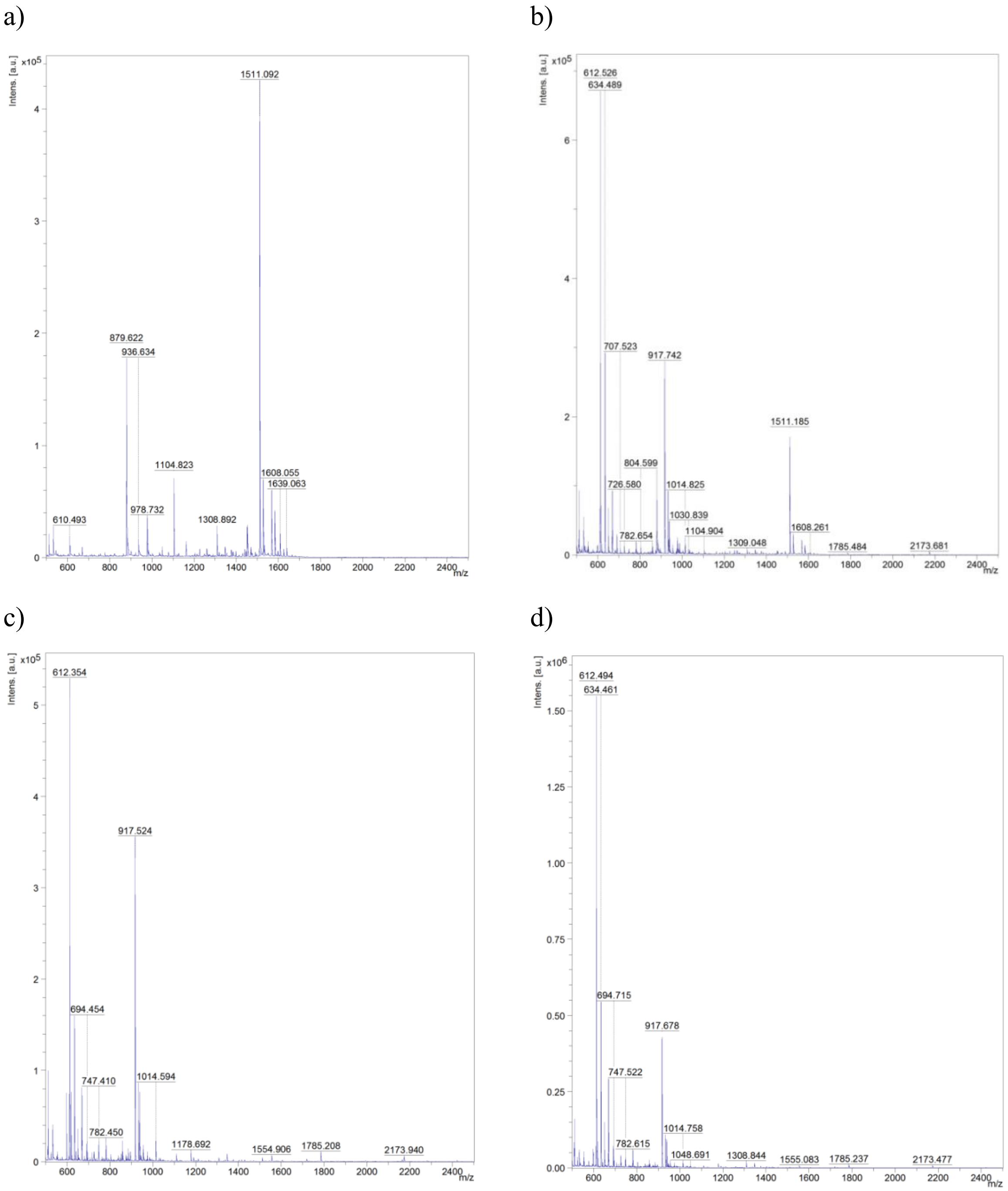

Fig. S5 MALDI–TOF mass spectra of peptide samples after incubation with matrix metalloproteinase 7. Observed peaks for ug52: (a) at the initial reaction time – ug52: (m/z) $[M+Na]^+$ = 1511.092; (b) after 1 h – ug52: (m/z) $[M+Na]^+$ = 1511.185; ug1: (m/z) $[M+H]^+$ = 612.526, (m/z) $[M+Na]^+$ = 634.489; (c) after 24 h – ug1: (m/z) $[M+H]^+$ = 612.526; (d) after 3 days – ug1: (m/z) $[M+H]^+$ = 612.526, (m/z) $[M+Na]^+$ = 634.489. Spectra are representative and were used for fragment identification rather than quantitative release analysis

Stability of ug51 under different pH and temperature conditions

The stability of peptide ug51 was evaluated in aqueous environments of varying pH (pH 7.2, deionized water; pH 6.6, HEPES buffer) and temperatures (37 °C and 40 °C) over a 7–day incubation period (Fig. S6). Quantitative HPLC analysis revealed a pronounced effect of pH and temperature on peptide stability. Ug51 showed higher stability at pH 7.2 than at pH 6.6. After 7 days, the remaining peptide content exceeded 60% in pH 7.2 conditions, compared to ~40% in pH 6.6 at 40 °C. Elevated temperature accelerated degradation in both pH environments,

with the most pronounced effect observed at pH 6.6 and 40 °C. These results indicate that acidic conditions significantly promote the degradation of ug51, while thermal acceleration of hydrolysis is secondary but additive to the pH effect.

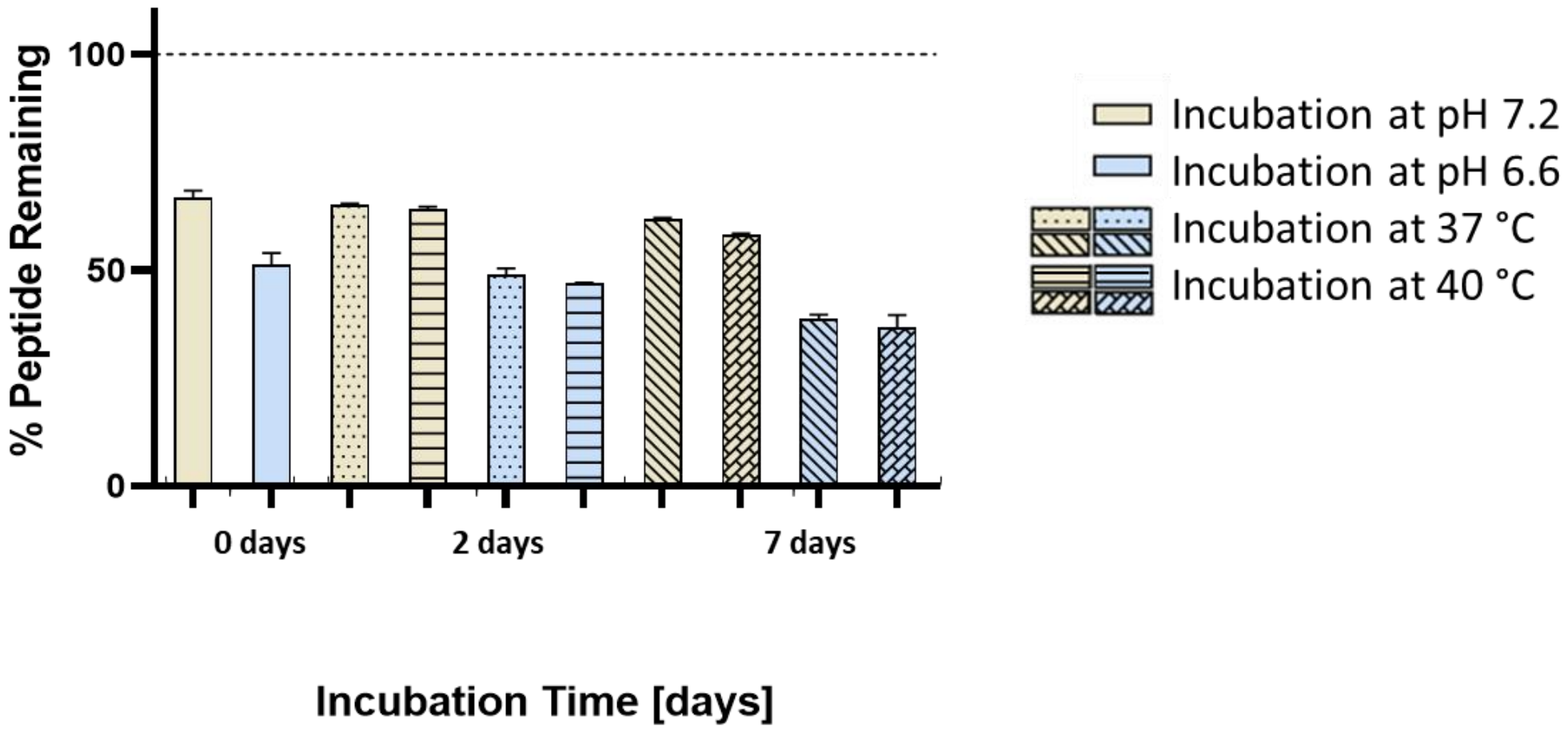

Fig. S6 Comparison of the percentage of remaining ug51 after incubation under different pH and temperature conditions

Cytocompatibility of ug51 and ug52 peptides in L929 fibroblasts and hFOB 1.19 osteoblasts (MTT assay)

In the MTT assay, both peptides were largely cytocompatible across the tested concentration ranges, as indicated by cell metabolic activity values remaining at or above the 70% ISO 10993-5 threshold for most conditions (Fig. S7 a and b). For ug51 (0.78 - 1600 µg/mL), hFOB 1.19 osteoblasts maintained metabolic activity consistently above the cytocompatibility threshold throughout the entire dose range (Fig. S7 a). In L929 fibroblasts, ug51 also preserved metabolic activity ≥70% across low-to-intermediate concentrations; however, a dose-dependent decline was observed at the upper end of the range, culminating in a reduction below 70% at 1600 µg/mL, indicating cytotoxicity at the highest dose tested in this cell line (Fig. S7 a). For ug52 (0.49 - 1000 µg/mL), L929 fibroblasts exhibited metabolic activity close to control levels across the full concentration range, remaining clearly above the 70% threshold even at 1000 µg/mL (Fig. S7 b). In contrast, hFOB 1.19 cells showed revealed metabolic activity ≥70% up to 500 µg/mL, followed by a marked decrease below the 70% threshold at 1000 µg/mL, suggesting reduced cytocompatibility of ug52 at the highest concentration specifically in osteoblasts (Fig. S7 b).

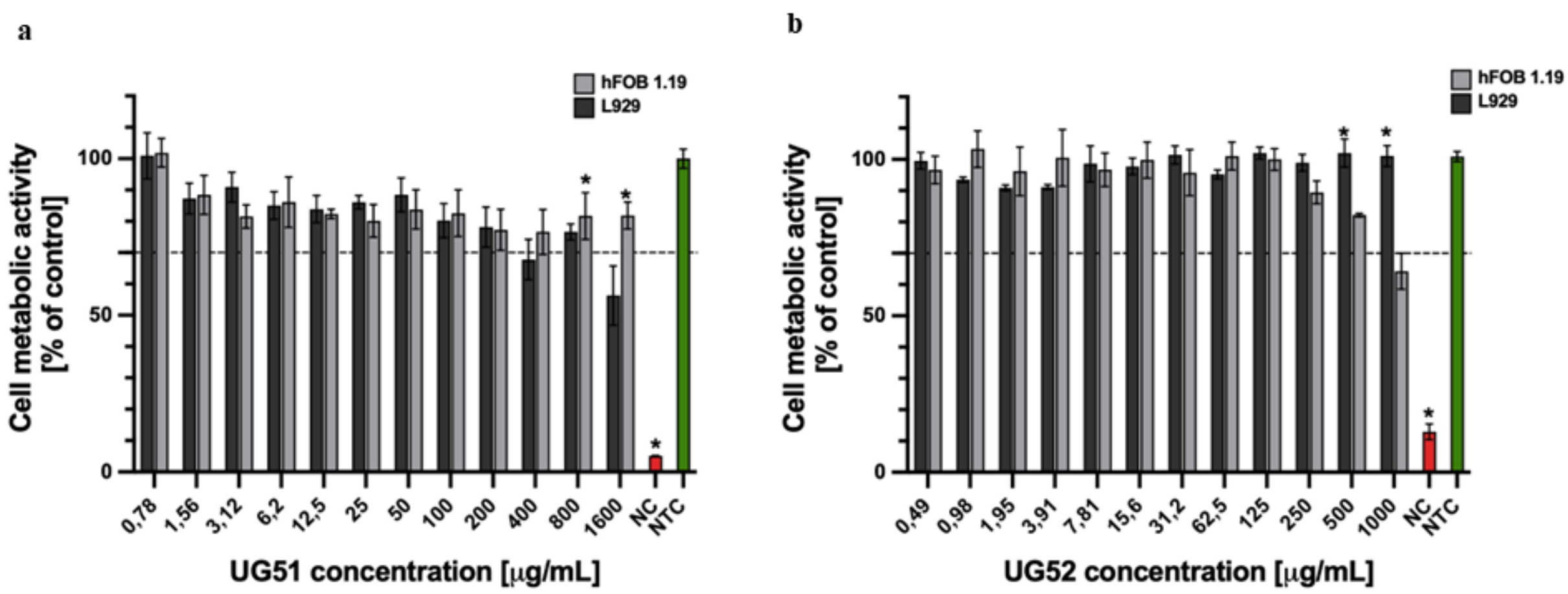

Fig. S7 Cytocompatibility of ug51 and ug52 in L929 fibroblasts and hFOB 1.19 osteoblasts assessed by the MTT assay. (a) Cell metabolic activity after 24 h exposure to ug51 (0.78 - 1600 µg/mL). (b) Cell metabolic activity after 24 h exposure to ug52 (0.49 - 1000 µg/mL). Results are expressed as a percentage of the non-treated control (NTC). The dashed line indicates the 70% threshold for cytocompatibility according to ISO 10993-5. NC, negative (cytotoxic) control; NTC, non-treated control. Data are presented as mean ± SEM (n > 6)
